# AI Versus Human Feedback in Gamified Color Education: A Four-Arm Cluster Randomized Controlled Trial

**DOI:** 10.3390/bs16071247

**Published:** 2026-07-22

**Authors:** Xiheng Shao, Fulong Liu, Jiao Wu, Mageswaran Sanmugam

**Affiliations:** 1 Centre for Instructional Technology and Multimedia, Universiti Sains Malaysia, Jalan Universiti, Gelugor 11700, Pulau Pinang, Malaysia; 2 School of Arts, Universiti Sains Malaysia, Jalan Universiti, Gelugor 11700, Pulau Pinang, Malaysia

**Keywords:** AI formative assessment, gamification, metacognitive regulation, cluster randomized controlled trial, moderated mediation, color education

## Abstract

Empirical evidence for AI formative assessment combined with gamification remains scarce in subjective aesthetic disciplines. This study examined whether such integration enhances metacognitive regulation in undergraduate color education and whether the effect is conditional on learners’ prior AI credibility belief. A four-arm, four-wave cluster-randomized controlled trial (N = 540, 24 classes, eight cross-allocated teachers) compared gamification with AI feedback, gamification with dose-matched human feedback, gamification only, and traditional instruction; the primary outcome was metacognitive regulation measured 4 weeks post-intervention. Gamification produced a small-to-medium effect over traditional instruction (*d* = 0.34, *p*_Holm = 0.015). AI and human feedback did not differ on average (*d* = 0.07; underpowered). Autoregressive mediation confirmed metacognitive regulation as a longitudinal mediator of intervention effects on motivation and achievement. On an exploratory basis, prior AI credibility belief moderated the AI-versus-human indirect effect on motivation (*p*_Holm = 0.034) but not achievement (*p*_Holm = 0.054), with higher-belief learners benefiting; given the limited power of the AI-versus-human comparison, this conditional pattern is reported as a tentative finding rather than as confirmatory evidence. These findings extend self-determination theory and the self-regulated learning framework to aesthetic education and provide preliminary support for the deployment of prior-trust-aware AI feedback, pending replication.

## 1. Introduction

Artificial intelligence has rapidly penetrated educational practice over the past several years, evolving from a peripheral classroom aid into a core element of learning assessment, process-level feedback, and personalized guidance (Holmes & Tuomi, 2022; Zawacki-Richter et al., 2019). Gamification has accumulated a parallel body of empirical evidence as an independent educational innovation, with meta-analytic support for consistent positive effects on learning across disciplines and educational levels (Zeng et al., 2024). Theoretically, the integration of these two pathways into a hybrid intervention should activate self-regulated learning (SRL) at two complementary levels: motivational engagement, externalized through gamification’s outcome-level feedback, and cognitive regulation, supported by AI-generated process-level signals. Empirical evidence for hybrid interventions, however, has been concentrated in STEM disciplines and general undergraduate populations; the aesthetic disciplines, where evaluative subjectivity is high, remain substantially under-represented.

Color education provides a particularly informative context for testing this integration. The evaluation of color work proceeds simultaneously at cognitive and affective levels: technical dimensions such as luminance contrast, harmony, and compositional balance can be assessed in a structured way, but dimensions such as color harmony perception, emotional expression, and aesthetic intent carry inherent evaluative subjectivity (Albers, 1963; Itten, 1970). This dual-loop characteristic places metacognitive regulation—learners’ planning, monitoring, and evaluation of their own learning—at the center of the transmission pathway: when external evaluation is uncertain, learners must rely more heavily on internal regulatory systems to adjust their learning strategies. Color education, therefore, constitutes a critical context in which to test whether AI formative feedback can augment metacognitive regulation in subjective aesthetic domains. AI formative feedback is particularly suited to this context because it can supply structured, dimension-complete, and immediately available process commentary at a frequency that expert human evaluation can rarely sustain in studio-style color courses, furnishing the external evaluative signal on which metacognitive monitoring depends precisely where such signals are otherwise scarce.

The central problem motivating the present study concerns the absence of evidence on whether integrating gamification with AI formative assessment benefits a subjective aesthetic discipline, and on whether any apparent AI benefit is specific to AI rather than a non-specific consequence of supplying additional process feedback; empirical evidence for such integration in color education is essentially absent, and the predominant two-arm designs comparing AI feedback to no-feedback or to traditional instruction cannot disentangle an AI-specific contribution from the general increment that any process-feedback source would produce (Banihashem et al., 2022; Cavalcanti et al., 2021). Three further considerations, subordinate to this central problem, shape the design. A temporal-identification consideration arises because most mediation studies use cross-sectional or two-wave within-intervention designs that do not satisfy the conditions for longitudinal mediation causal identification, namely that X has ceased before M is measured and that M is measured before Y (Cole & Maxwell, 2003; Maxwell et al., 2011). A design consideration arises because small cluster numbers and teacher–treatment confounding remain prevalent in cluster trials and have rarely been addressed together. A heterogeneity consideration arises because average treatment effects are typically reported without testing whether learners’ prior beliefs generate substantive effect heterogeneity, even though the interaction between AI formative feedback and prior AI credibility belief constitutes an open theoretical question (Komiak & Benbasat, 2006; Lankton et al., 2015). The overarching research question is whether integrating gamification with AI formative feedback enhances metacognitive regulation in color education, and whether any AI-specific contribution to this effect is conditional on learners’ prior AI credibility beliefs.

To address this central problem and its subordinate considerations, a four-arm, four-wave cluster randomized controlled trial was conducted. The design separates the AI-specific contribution from the general process-feedback increment through a dose-matched active control, introduces prior AI credibility belief as a first-stage moderator, cross-allocates eight teachers across arms to disentangle teacher from treatment effects, and measures the primary outcome four weeks after intervention cessation to meet the conditions for longitudinal mediation identification.

The remainder of the article is organized as follows: Section 2 develops five hypotheses; Section 3 describes the four-arm × four-wave cluster RCT; Section 4 presents results; Section 5 interprets findings against meta-analytic benchmarks, causal identification, and heterogeneity considerations; Section 6 summarizes contributions and future directions.

## 2. Literature Review and Hypotheses

### 2.1. Gamification and Metacognitive Regulation Through the SDT–SRL Integration Framework

Gamification in education refers to the use of game design elements such as points, badges, levels, progress dashboards, and unlocking pathways in non-game instructional settings and has been examined across subject areas and educational levels, with generally positive but heterogeneous effects on engagement and achievement. Gamification’s effect on learning has accumulated meta-analytic support: *g* = 0.49 for cognitive learning (Sailer & Homner, 2020), composite *g* = 0.504 across 30 studies (Bai et al., 2020), and *g* = 0.782 across 22 experimental studies (Zeng et al., 2024). Consistent replication supports gamification’s effectiveness, but evidence on its transmission mechanisms remains fragmented.

Self-determination theory (Ryan & Deci, 2020) explains gamification from an intrinsic-motivation perspective: core mechanics—points, badges, progress dashboards—satisfy competence needs and sustain engagement. SDT alone explains why learners remain engaged, but not how engagement translates into learning outcomes. Self-regulated learning theory (Schunk & Greene, 2018; Zimmerman, 2008) posits that when competence needs are satisfied, learners activate metacognitive regulation—planning, monitoring, and evaluating—which directly shapes knowledge construction and skill development (Dignath & Büttner, 2008; Pintrich, 2004). The SDT–SRL integration provides the theoretical foundation for the gamification-to-outcomes pathway. Within this integrated account, gamification mechanics and process feedback act on complementary points along a single chain: the former sustaining competence-based engagement, and the latter supplying the external evaluative information that metacognitive monitoring requires, so that activated regulation converts sustained engagement into the knowledge construction underlying subsequent motivation and achievement.

**Hypothesis** **1 (H1).** *Learners receiving the gamification intervention (Arm C) will demonstrate significantly greater T3 metacognitive regulation than learners receiving traditional instruction (Arm D).*

### 2.2. Feedback-Source Effects: Separating AI Specificity from General Process Increment

Formative feedback consistently supports metacognitive regulation. Hattie and Timperley’s (2007) three-level framework identifies process feedback as particularly effective for activating metacognitive regulation. AI formative feedback has emerged as a new process-feedback source. Systematic reviews of automated and learning-analytics feedback in higher education describe systems that deliver timely, scalable, and dimensionally structured commentary while reporting inconsistent effects and few controlled comparisons that isolate the AI source itself (Banihashem et al., 2022; Cavalcanti et al., 2021); most such studies have used two-arm designs comparing AI to no-feedback or traditional instruction, conflating two distinct contributions: the overall increment of process feedback (which any source can produce) and the AI-specific contribution (structural advantages in structuring, dimensional coverage, immediacy, and dose stability).

The present study decomposes this identification into two layers. The first addresses AI specificity: AI feedback offers structural advantages in structuring, dimensional coverage, sustained immediacy, and dose stability, whereas human feedback offers context-sensitivity, interpersonal warmth, and empathic communication.

**Hypothesis** **2a (H2a).** *With feedback doses strictly matched across four dimensions—frequency, dimension count, word-count range, and timing—AI formative feedback (Arm A) will differ from human teaching assistant process feedback (Arm B) in T3 metacognitive regulation. Direction is not predicted; the contrast is two-sided.*

The second layer addresses the overall feedback-source increment: process feedback supplements gamification’s outcome-level signals and should yield a metacognitive increment regardless of source.

**Hypothesis** **2b (H2b).** *Process feedback added to gamification (Arms A and B combined) will produce significantly greater T3 metacognitive regulation than gamification alone (Arm C).*

### 2.3. Metacognitive Regulation as a Longitudinal Mediator (T0 → T1 → T3)

The classical SRL chain linking metacognition, self-efficacy, and performance is supported in cross-sectional studies (Pintrich, 2004; Schunk & Greene, 2018), but cross-sectional and two-wave within-intervention models tend to overestimate indirect effects because Y is measured while X is still being administered. Genuine longitudinal mediation causal identification requires that X-administration has ended, M is measured after X, and Y is measured after M (Cole & Maxwell, 2003; Maxwell et al., 2011).

The four-wave structure—T0 baseline → T1 (week 9) → T2 (week 14, end of intervention) → T3 (week 18, four weeks post-intervention)—positions T3 after X has ceased, enabling T0 → T1 → T3 to satisfy this identification condition.

**Hypothesis** **3 (H3).** *T1 metacognitive regulation will mediate the longitudinal effect of intervention condition on T3 learning motivation and T3 color-work achievement, with indirect effects remaining significant after T0 covariate adjustment.*

### 2.4. Prior AI Credibility Belief as a First-Stage Moderator

H2a addresses the AI-vs-human average effect but abstracts away from a theoretically expected source of effect heterogeneity: learners’ prior credibility beliefs about AI systems. Technology-acceptance research shows that learners’ prior trust shapes cognitive acceptance and adoption of system outputs (Komiak & Benbasat, 2006; Lankton et al., 2015). In the AI formative-feedback context, high-trust learners are more likely to integrate AI feedback into metacognitive monitoring, whereas low-trust learners may adopt defensive skepticism, attenuating this effect. If such heterogeneity is present, the average-level H2a effect should be offset by opposing subgroup contributions, approaching zero, with high-trust learners in Arm A showing significantly higher T3 metacognitive regulation than their Arm B counterparts.

We adopt Hayes (2015) Model 7 rather than Model 8: the theoretical assumption is that prior trust shifts whether learners integrate AI feedback into metacognitive monitoring (X → M), without comparable moderation on the X → Y direct path. Model 8 is fitted as a planned robustness check.

**Hypothesis** **4 (H4).** *Prior AI credibility belief will moderate the indirect effect of Arm A (vs. B) on T3 outcomes through T1 metacognitive regulation. The conditional indirect effect will be significantly positive at high belief and near zero at low belief; the index of moderated mediation will have a 95% CI excluding zero.*

### 2.5. Hypothesis Model Summary

**Figure 1:**
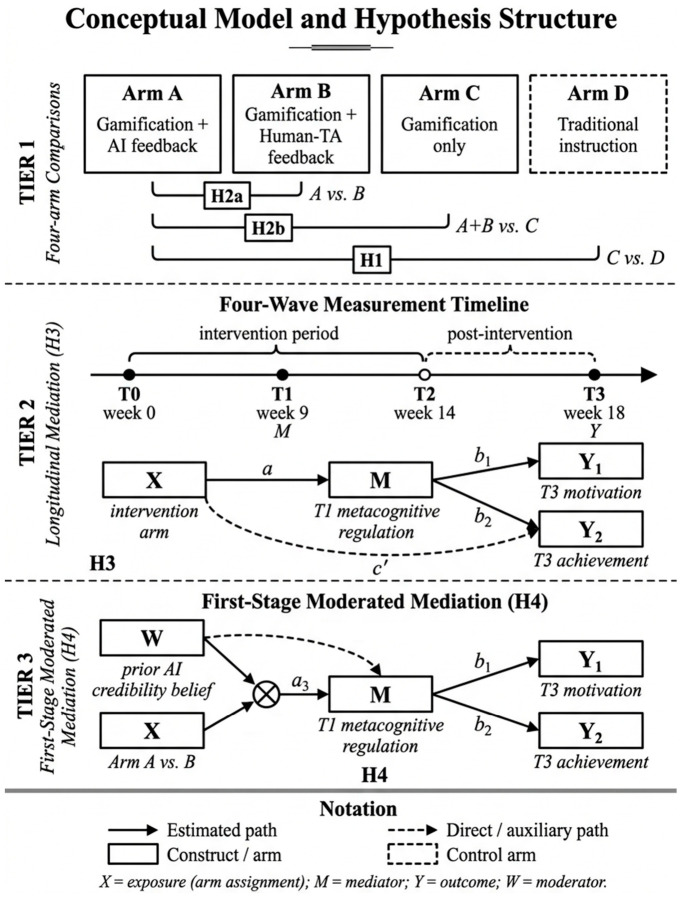
Conceptual model and hypothesis structure.

The five hypotheses form a three-tier structure: the top tier comprises the four-arm comparisons (H1, H2a, H2b); the middle tier addresses full-sample longitudinal mediation (H3); the bottom tier addresses first-stage moderated mediation (H4). Figure 1 presents the conceptual model and hypothesis structure across these three tiers. The three tiers differ in evidential strength, a distinction carried through to interpretation: H1 and H3 rest on well-replicated meta-analytic and self-regulated-learning foundations and are treated as confirmatory, whereas the AI-specificity contrast embedded in H2a and the first-stage moderated mediation in H4 rest on more tentative theoretical grounds and are framed as exploratory throughout. Table 1 summarizes the five hypotheses, their analytic strategies, methods, and a priori power targets.

The three-tier structure organizes the five hypotheses. Tier 1 (top) presents the four-arm comparisons: H1 (gamification vs. traditional instruction, C vs. D), H2a (AI feedback vs. dose-matched human feedback, A vs. B), and H2b (pooled process feedback vs. gamification alone, A + B vs. C). Tier 2 (middle) presents the four-wave measurement timeline (T0/T1/T2/T3) and the longitudinal mediation pathway from intervention exposure (X) to T1 metacognitive regulation (M) to T3 motivation (Y_1_) and T3 achievement (Y_2_); T3 is positioned four weeks after intervention cessation to enable longitudinal mediation causal identification (H3). Tier 3 (bottom) presents the first-stage moderated mediation framework in which prior AI credibility belief (W) moderates the indirect effect of A versus B through T1 metacognitive regulation (H4). Solid arrows represent estimated paths; dashed arrows represent direct or auxiliary paths. The dashed border around Arm D indicates the no-intervention control condition.

## 3. Methods

### 3.1. Design, Pilot Study, and Participants

We conducted a cluster-randomized controlled trial across color composition and color design courses for design undergraduates at a Chinese university, using a 4-arm × 4-wave design. The study protocol, hypotheses, and analysis plan were finalized and internally documented prior to data collection, and raw data and analysis code will be made publicly available upon publication. The study was approved by the institutional research ethics committee 2024-XJU-EDU-038. Participants provided written informed consent. As an ethical equivalence measure, students in the control arm (D) received a supplementary workshop covering the Arm A intervention components after the end of the semester.

The sampling frame comprised all intact classes scheduled to take the color composition and color design courses within the design school during the study semester, of which 31 were assessed for eligibility. Restricted randomization (Moulton, 2004) was used at the cluster level. Seven of these classes were excluded for the administrative reasons reported by arm in Table 2 (teacher maternity leave, teacher reassignment, high student-transfer rates, class-size deviation outside the 21–25 range, and timetable conflict). The remaining 24 classes were assigned via a computer-generated random sequence, subject to the constraint that each of the eight teachers taught at most three classes covering at least two intervention conditions, with non-conflicting time slots. Of 184 feasible allocation schemes enumerated in advance, one was drawn. The realized scheme was drawn from this enumerated feasible set after removal of the seven excluded classes, with the complete eight-teacher cross-arm allocation matrix and the arm-coverage constraints documented in Supplementary S2. The restricted randomization was constrained solely by administrative feasibility and did not balance baseline variables post hoc; baseline p-values therefore reflect the true sampling distribution.

Baseline (T0) measurement was completed after cluster-level randomization but before student-level unmasking, which was performed immediately before the first week of classes. Unmasking disclosed only the student’s own assigned condition.

An eight-week pilot (*n* = 46, two classes) verified the procedural pipeline (Supplementary S0). The four arms were gamification with AI formative feedback (A); gamification with human teaching assistant process feedback (B; active control); gamification only (C); and traditional instruction (D). The four measurement waves were T0 (baseline, one week before intervention onset), T1 (week 9, primary mediator measurement), T2 (week 14, end of intervention; used for descriptive purposes and mediation robustness), and T3 (week 18, four weeks after intervention cessation; primary outcome). Setting T3 four weeks post-intervention satisfied the longitudinal mediation identification condition, as X was no longer administered when Y was measured. The 14-week duration aligned with the 8–12-week window for SRL metacognitive effects (Dignath & Büttner, 2008).

Participants were 549 students nested within 24 intact classes (six per arm; class sizes ranged from 21–25). Anticipated cumulative attrition at T3 was 18%, yielding an expected primary analysis sample of approximately 449. Table 2 presents the participant flow following the CONSORT extension for cluster-randomized trials (Campbell et al., 2012). With 24 level-2 units, the design fell below the conventional 30+ threshold for stable random-effects variance-component estimation in multilevel models (McNeish & Stapleton, 2016); we therefore did not estimate random effects and instead relied on cluster-robust standard errors, supplemented by wild-cluster bootstrap (Cameron et al., 2008).

Three unanticipated implementation events occurred over the intervention. One Arm A class had a four-day feedback delay in week 11 due to a sports-day timetable adjustment; one Arm B teaching assistant was absent for three days in week 14 with backup-delivered feedback; one Arm A class experienced a five-hour AI-system outage in week 7 during scheduled network maintenance, with feedback delivered via backup API (median response time temporarily 18 → 32 h). None constituted a protocol deviation; sensitivity analyses excluding affected classes did not alter principal conclusions (Supplementary S1).

### 3.2. Intervention Protocols, Teacher and Teaching Assistant Allocation, and Implementation Fidelity

Strict matching of feedback dose on four dimensions was central to the identification validity of H2a. Arms A and B were matched on feedback frequency (weekly, with feedback returned within 48 h of submission), feedback dimension count (five fixed dimensions: color harmony, luminance contrast, color-emotion expression, composition and balance, and technical execution), feedback word-count range (200–400 characters), and feedback timing. The two arms differed only in the feedback source: Arm A feedback was generated by the ERNIE 4.0 large language model using a structured system prompt, whereas Arm B feedback was written by trained human teaching assistants using the same five-dimensional template. Students in Arms A and B were informed of the source of their process feedback, that is, whether it was generated by an AI system or written by a human teaching assistant; this disclosure status bears on the interpretation of feedback uptake and is revisited in Section 5.4.

Matching the four dose dimensions equalizes the quantity and structural coverage of feedback. However, it does not guarantee equivalence in qualitative properties such as tone, contextual sensitivity, and responsiveness to a learner’s expressed intention, which remain plausible sources of difference between automated and human sources. Illustrative feedback examples for each arm, showing the shared five-dimension structure alongside these residual stylistic differences, are provided in Supplementary Table S3; the qualitative consequences of this distinction are taken up in Section 5.4.

The course syllabus required one color design assignment per week. Week 1 was an introductory week without an assignment; assignments were issued, submitted, and returned weekly from week 2 through week 14, with 13 cycles of formal feedback in total. T1 measurement took place at the start of week 9, by which point students had received a cumulative total of seven feedback cycles.

Gamification mechanics, common to Arms A, B, and C, comprised stage-based points, an assignment–skill unlocking pathway, a class-level progress dashboard, stage-based badges, and an individual portfolio archive. Arm C received the gamification mechanics without any process feedback and served as the critical comparison for separating the H1 gamification main effect from the H2b overall process-feedback increment. Arm D followed an equivalent course outline and contact hours but received neither gamification mechanics nor process feedback. Table 3 compares the intervention components across the four arms.

#### 3.2.1. Teacher Selection and Cross-Arm Allocation

Eight in-house teachers participated (rank ≥ lecturer, ≥5 years’ experience, having taught equivalent courses in the most recent 3 years, and having completed a 32 h preparation workshop covering all four-arm protocols). Each teacher taught three classes. Cross-arm allocation followed two constraints: each teacher covered at least two conditions (ensuring teacher × treatment identifiability), and each arm was covered by at least four teachers (avoiding teacher–treatment collinearity). The full 8 × 24 allocation matrix is provided in Supplementary Table S2. Teachers were assigned only to arms in which they passed an arm-specific competence assessment.

#### 3.2.2. Teaching Assistant Structure for Arm B

Six teaching assistants (graduate students at the design school, with 24 h of feedback-writing training) were assigned one-to-one to the six Arm B classes without rotation throughout the intervention. Teaching assistants provided only process feedback; they did not participate in classroom teaching, terminal grading, or the points system.

#### 3.2.3. Implementation Fidelity

Implementation fidelity was secured through teacher training logs, platform back-end logs, biweekly classroom observations, and control-arm purity checks. Cumulative feedback delivery rates by T2 were 95.7% (Arm A) and 91.4% (Arm B), consistent with the 5–10% AI-versus-human delivery gap in the literature (Banihashem et al., 2022). The lowest within-class delivery rates were 88.6% (Arm A, network outage) and 84.2% (Arm B, family emergency). Classroom observation ratings ranged 3.7–4.5 across T1/T2/T3, with inter-observer correlations r = 0.61–0.84. The intraclass correlation among Arm B teaching assistants was 0.66 (95% CI [0.38, 0.82]), though precision was limited by the small sample size (*n* = 6). Nine passive contamination events (three Arm A, two Arm B, one Arm C, three Arm D) were recorded; sensitivity analyses excluding affected students did not alter principal conclusions.

### 3.3. AI Evaluation System, Credibility-Belief Measurement, and Four-Wave Measurement

Arm A AI formative feedback was generated by ERNIE 4.0 via Baidu AI Cloud’s enterprise API under an SLA fixing the model version at ERNIE-4.0-8K-0613 throughout the intervention period (excluded from server-side auto-upgrades). Generation parameters were temperature = 0, top-*p* = 1; each rating was an independent context-free call with structured JSON output. Version identifiers, timestamps, and raw responses were persistently archived.

AI-feedback reliability was established at three independent levels: agreement with three independent color-education experts (ICC [2,k] on *n* = 60, target ≥ 0.75), two-week test–retest stability (*n* = 40), and intra-author consistency (Zheng et al., 2023). A critical boundary condition was that ERNIE 4.0 functioned only as Arm A’s process-feedback source and was excluded from T3 outcome assessment, eliminating circular contamination by design.

**Figure 2:**
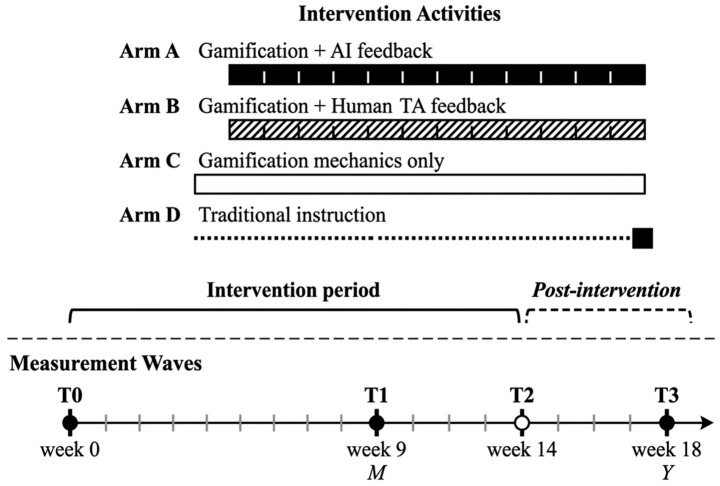
Measurement and intervention timeline.

T0 measurement was completed one week before intervention onset, after cluster-level randomization but before student-level unmasking. T0 instruments included the Chinese MAI short form (12 items; Schraw & Dennison, 1994; Liu & Liu, 2010), the motivation subscales of the Chinese MSLQ (15 items; Pintrich et al., 1993), a 10-item color-knowledge pretest, a 4-item prior AI credibility belief scale adapted from the functionality and reliability subscales of Lankton et al. (2015) (full items and selection criteria in Supplementary S5; Cronbach’s α = 0.81 in pilot, CVI = 0.83), three manipulation-check items, and demographic variables. T1 added an 8-item learning-engagement scale; T2 retained MAI + engagement; T3 included MAI, MSLQ, color-knowledge posttest, and color-work achievement assessment. Figure 2 presents the full measurement and intervention timeline.

Intervention Activities (top) shows each arm’s activity pattern: Arms A and B received 13 weekly assignment cycles with AI or dose-matched human teaching assistant feedback (weeks 2–14); Arm C had gamification mechanics only (weeks 1–14, no process feedback); Arm D received traditional instruction with a single end-of-term assignment at week 14. Solid bracket = intervention period; dashed bracket = four-week post-intervention follow-up. Measurement Waves (bottom) shows the four assessment points: T0 (baseline), T1 (mediator), T2 (end of intervention, hollow circle), and T3 (primary outcome, four weeks after intervention cessation). Detailed instruments at each wave are reported in Section 3.3.

The primary mediator for H3 and H4 was the 6-item MAI cognitive-regulation subscale at T1, with the 12-item total score as a planned sensitivity analysis. T3 color-work achievement was assessed on two parallel tracks. The primary outcome was the consensus rating of three independent color-education experts not involved in intervention delivery and without contact with ERNIE 4.0, using a rubric structurally aligned with the AI prompt dimensions but independently worded; rating-batch order was randomized, and batches were spaced 2–3 days apart, with target ICC(2,3) ≥ 0.75, and disagreements resolved in consensus meetings. As a secondary descriptive measure, GPT-4o and Claude 3.5 served as independent LLM raters for robustness comparison.

### 3.4. Analytic Strategy and Statistical Power

Primary analyses used Mplus 8.10 with TYPE = COMPLEX, class as cluster variable, the MLR estimator, and cluster-robust sandwich standard errors (McNeish & Stapleton, 2016). Teacher dummies were included as level-2 covariates. The eight-teacher cross-arm allocation, in which each teacher’s three classes were assigned across three different arms, satisfied the 24-of-32 cell-coverage constraint and identified marginal teacher and treatment effects separately; the full teacher × treatment interaction was constrained and is reported in restricted form as a sensitivity analysis.

H1 (C vs. D), H2a (A vs. B), and H2b (A + B vs. C) were tested with cluster-robust ANCOVA. All three contrasts employed two-sided tests; H1 and H2b carried directional theoretical predictions (H1: gamification > traditional; H2b: process feedback > gamification alone, following Hattie & Timperley, 2007), interpreted as requiring the predicted sign of Δ_adj for confirmatory interpretation while keeping the statistical decision rule two-sided. Degrees of freedom followed the Bell and McCaffrey (2002) small-cluster correction (G − *p*; G = 12 for H1 and H2a, 18 for H2b).

H3 was tested with a four-wave autoregressive mediation model (T0 → T1 → T3) following Cole and Maxwell (2003). Covariates included T0 metacognitive regulation, T0 MSLQ, and T0 color-knowledge pretest. The T0 → T1 autoregressive path was estimated to control for M stability and avoid bias in the a-path estimate (Maxwell et al., 2011). Cross-arm equality of the autoregressive path was tested as a prerequisite assumption (rejection criterion: Δχ^2^ *p* < 0.05 or ΔCFI > 0.01). Indirect effects were computed using Monte Carlo confidence intervals with 20,000 resamples (Preacher & Selig, 2012).

H4 was tested using Hayes Model 7 (Hayes, 2015), restricted to the A and B subsamples (*n* = 273). The moderator W was standardized T0 prior to AI credibility belief; the X × W interaction was entered jointly with X and W as predictors of T1 metacognitive regulation, which in turn predicted T3 motivation and T3 achievement on two parallel outcome paths. Given only 12 clusters in this subsample, cluster wild bootstrap (Cameron et al., 2008; 5000 resamples; 6-point Webb (2023) distribution) served as the primary inference method, with bias-corrected ordinary bootstrap as a secondary reference. Hayes Model 8 (W moderating X → Y direct paths) was fitted as a planned robustness check.

For the achievement path, the T0 color-knowledge pretest served as a proxy for the Y baseline. Because the pretest construct (declarative knowledge) is not fully aligned with the T3 color-work achievement construct (production capability), a planned robustness check fitted the achievement-path mediation model with and without the T0 pretest; principal conclusions were reported only if directions were consistent and 95% CIs overlapped at ≥80%.

Three sensitivity-analysis schemes were run in parallel: wild-cluster bootstrap, class-fixed effects dummy specification, and MAI 12-item total-score mediation. Multiple-comparison correction used the Holm (1979) step-down procedure independently within three families: H1/H2a/H2b (k = 3); the two H3 paths (k = 2); and the two H4 indices of moderated mediation (k = 2). Both raw and Holm-corrected *p*-values are reported.

A priori power was calculated for the post-attrition analytic samples (Table 4) under cluster-robust ANCOVA with α = 0.05 and target power = 0.80. Power was estimated for both the FIML primary sample (N = 540) and the complete-case sensitivity sample (N = 456), across three intracluster correlation (ICC) scenarios (0.05, 0.10, and 0.15; the baseline ICC = 0.10, following Hedges & Hedberg, 2007).

## 4. Results

The results are organized so that the principal outcome for each hypothesis is stated before the supporting technical detail. Gamification produced a small but identifiable advantage over traditional instruction on metacognitive regulation (H1); AI and dose-matched human process feedback did not differ on average within the available power (H2a); pooled process feedback exceeded gamification alone only marginally and sensitively to specification (H2b); T1 metacognitive regulation mediated the longitudinal effect of the intervention on T3 motivation and achievement (H3); and prior AI credibility belief conditioned the AI-versus-human indirect effect on the motivation outcome, with the achievement-outcome moderation not reaching significance (H4). The subsections that follow report sample characteristics and baseline equivalence (Section 4.1), manipulation and dose checks (Section 4.2), measurement models (Section 4.3), the main-effect contrasts (Section 4.4), longitudinal mediation (Section 4.5), and moderated mediation (Section 4.6), with a consolidated numerical recapitulation in Section 4.7.

### 4.1. Sample Characteristics, Attrition, and Baseline Equivalence

Primary analyses used FIML on N = 540 (A = 140, B = 133, C = 138, D = 129); complete-case sensitivity analyses on N = 456 yielded directionally consistent conclusions.

Cluster-robust ANCOVAs on 10 baseline variables revealed two marginal differences: prior color-course experience (*p* = 0.087; Arm D slightly lower) and T0 learning motivation (*p* = 0.043; Arm C slightly higher than Arm D). Both were addressed through covariate adjustment, with unadjusted models reported as sensitivity references (Supplementary Table S5). The remaining eight variables were equivalent (*p* = 0.13–0.91). The unconditional ICC for T0 metacognitive regulation was 0.083 (95% CI [0.041, 0.142]), at the lower end of typical educational-research ranges (Hedges & Hedberg, 2007) and slightly below the methods-baseline ICC = 0.10. Prior AI credibility belief had a class-level ICC of 0.041 and was treated as individual-level in H4.

Cumulative T3 attrition was 84 students (15.6%), below the 18% projected. Little’s MCAR test was non-significant, χ^2^(48) = 56.71, *p* = 0.184. Dropouts had significantly lower T0 learning motivation than completers (M_drop = 3.42 vs. M_retain = 3.71, *p* = 0.003); other variables did not differ. T0 learning motivation was therefore included as a FIML auxiliary variable. Sample characteristics, baseline equivalence, and intraclass correlations are reported in Table 5.

### 4.2. Manipulation Check

The manipulation check operated at three levels: behavioral facts, perceived intensity, and dose matching.

**Behavioral facts.** Platform back-end logs documented substantial between-arm differences. Arms A and B had cumulative feedback delivery rates of 95.7% and 91.4%; Arm C received zero process feedback but had a median platform access of 3.2 visits/week (Arms A: 3.4; B: 3.3); Arm D had no platform access.

**Perceived intensity.** Cluster-robust ANCOVA results for the three T1 manipulation-check items are summarized in Table 6.

**Dose matching.** Arms A and B were matched on all four dose dimensions: median feedback word counts differed by 12 characters (IQR overlap 86%), dimension-coverage differed by 3.3% (96.7% vs. 93.4%), feedback timing was within 48 h in both arms (medians 18 h vs. 22 h), and keyword Jaccard overlap was 0.78. The metacognitive difference between Arms A and B, therefore, cannot be attributed to dose and can be attributed only to feedback-source specificity.

### 4.3. Measurement Models and Common-Method Bias

Mplus CFAs indicated acceptable measurement fit: CFI 0.928–0.971; TLI 0.916–0.962; RMSEA 0.039–0.072 (the 0.072 value, for the MAI cognitive-regulation subscale, lies between the Hu and Bentler (1999) 0.06 and 0.08 cutoffs); SRMR ≤ 0.058; CR 0.74–0.89; AVE 0.48–0.66 (the 0.48 value is acceptable under Fornell and Larcker’s (1981) criterion that CR > 0.70 with AVE < 0.50, but only marginally); HTMT 0.62–0.86 (the 0.86 value, between MAI cognitive-regulation and MSLQ self-efficacy, lies between Henseler et al.’s (2015) strict 0.85 and lenient 0.90 cutoffs). The simultaneous AVE = 0.48 and HTMT = 0.86 on this scale-pair means discriminant validity is not strongly supported; we therefore interpret the metacognition–motivation indirect effect in H3 with caution and flag this as a measurement-level limitation in Section 5.5. Harman’s single-factor test indicated 26.4% variance, well below 50%. Marker-variable partial correlations were all below 0.13, indicating that common-method bias does not pose a substantive threat.

### 4.4. Main Effects: H1, H2a, and H2b

Cluster-robust ANCOVA results for T3 metacognitive regulation as the primary outcome are reported in Table 7.

**H1 (C vs. D).** Arm C (M_T3 = 3.78, SD = 0.69) significantly outperformed Arm D (M_T3 = 3.55, SD = 0.72), Δ_adj = 0.23 (95% CI [0.10, 0.36]), *d* = 0.34, *t*(10) = 3.65, *p*_raw = 0.005, *p*_Holm = 0.015. The direction of the gamification main effect is consistent with the meta-analytic estimate (Zeng et al., 2024); the observed effect size, while smaller than the meta-analytic composite (*g* = 0.78) and below the *d* = 0.50 a priori target used in the power calculation, was sufficient to reach significance in this sample. The contrast between observed *d* = 0.34 and meta-analytic *g* = 0.78 is consistent with the methods-stage expectation that aesthetic subjectivity in color education would attenuate gamification effects.

**H2a (A vs. B).** Arm A (M_T3 = 3.97) and Arm B (M_T3 = 3.92) did not differ significantly, Δ_adj = 0.05 (95% CI [−0.13, 0.23]), *d* = 0.07, *t*(10) = 0.61, *p*_raw = 0.555, *p*_Holm = 0.555. Following the prespecified power-boundary statement in Section 3.4, this null result cannot be interpreted as evidence for equivalence between AI and human feedback—the actual power for H2a was approximately 0.50. The result is more accurately described as “no average difference between AI and human process feedback was detected within the power range of the present study.” The H4 moderated mediation results in Section 4.6 provide a partial heterogeneity-based interpretation of this null average effect, although, as we report below, the H4 evidence is itself only partially supported.

**H2b (A + B vs. C).** The pooled Arms A and B (M_T3 = 3.95, *n* = 273) significantly outperformed Arm C (M_T3 = 3.78, *n* = 138), Δ_adj = 0.17 (95% CI [0.02, 0.32]), *d* = 0.24, *t*(16) = 2.49, *p*_raw = 0.024, *p*_Holm = 0.048. The overall increase in process feedback over gamification alone received marginal support, with p_Holm near the 0.05 threshold and the effect size in the small range. We explicitly state that “marginal” here means exactly that, and we caution against overinterpreting the result as a robust process-feedback advantage.

**Figure 3:**
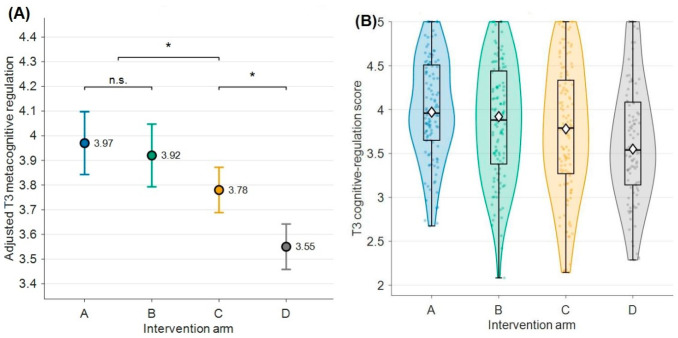
Adjusted T3 metacognitive regulation across the four arms. Panel (**A**) shows the covariate-adjusted T3 marginal means with 95% confidence intervals for Arms A, B, C, and D. Panel (**B**) shows the distribution of individual T3 cognitive-regulation scores by arm as overlaid violin and box plots with jittered individual data points, with the adjusted mean marked. Covariates are as in Table 7. Brackets above Panel A denote the Holm-corrected contrasts reported in Table 7 (H1, C vs. D; H2a, A vs. B; H2b, A + B vs. C). * Holm-corrected *p* < 0.05; n.s. = not significant.

The marginal means of T3 metacognitive regulation across the four arms (controlling for all covariates) followed the pattern A ≈ B > C > D, with values of A = 3.97, B = 3.92, C = 3.78, and D = 3.55. The adjusted-mean pattern and the underlying spread of individual scores across the four arms are displayed in Figure 3. Three sensitivity-analysis schemes (wild cluster bootstrap, fixed-effects dummy, and ignoring nesting) yielded H1 effect sizes of 0.30 to 0.37, non-significant H2a in all schemes, and H2b effect sizes of 0.20 to 0.27. One of the three H2b sensitivity schemes (class fixed-effects dummy) yielded *d* = 0.20, *p* = 0.062, attenuating to non-significance; the principal H2b conclusion should therefore be regarded as specification-sensitive (Supplementary Table S5).

Figure 3 makes the A ≈ B > C > D ordering directly visible and shows that the separation between the two process-feedback arms and gamification alone is modest relative to within-arm dispersion, consistent with the small adjusted contrasts and the specification sensitivity reported for H2b. Panel B shows substantial overlap of the individual-score distributions across arms, indicating that arm-level differences operate against a background of considerable individual variability rather than reflecting uniform shifts. This pattern frames the conditional, trust-dependent reading of the AI-versus-human comparison developed in Section 4.6.

### 4.5. H3 Longitudinal Mediation

**Figure 4:**
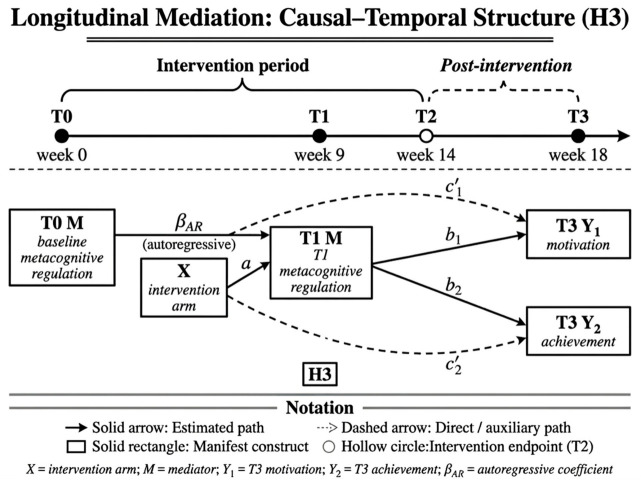
Longitudinal mediation: causal–temporal structure (H3). The upper layer shows the four-wave measurement timeline (T0/T1/T2/T3), with the solid bracket marking the intervention period and the dashed bracket marking the four-week post-intervention follow-up; T2 (hollow circle) marks intervention cessation. The lower layer shows the path diagram with five constructs aligned horizontally to their measurement waves: T0 metacognitive regulation (T0 M), intervention arm (X), T1 metacognitive regulation (T1 M, mediator), T3 motivation (Y_1_), and T3 achievement (Y_2_). Solid arrows mark estimated paths: the autoregressive path β_AR (T0 M → T1 M), the a path (X → T1 M), and the b paths (T1 M → Y_1_ as b_1_; T1 M → Y_2_ as b_2_). Dashed arrows mark the direct paths c′_1_ (X → Y_1_) and c′_2_ (X → Y_2_). Path coefficients with 95% CIs are reported in Table 7.

The four-wave T0 → T1 → T3 longitudinal mediation results are summarized in Table 7. Figure 4 presents the path diagram and causal–temporal structure.

**Prerequisite assumption.** Comparing the freely estimated and equality-constrained models for the T0 → T1 metacognition autoregressive path yielded Δχ^2^(3) = 4.27, *p* = 0.234, and ΔCFI = 0.003, neither of which reached the prespecified rejection thresholds; the cross-arm equality assumption was therefore retained. Primary H3 inference used the constrained model, with a common autoregressive coefficient of β_AR = 0.582 (95% CI [0.521, 0.638]) across the four arms, indicating that the intervention shifted the level of metacognitive regulation rather than its stability structure.

**Indirect effects.** The motivation-path indirect effect was ab_1_ = 0.083 (95% CI [0.039, 0.135], Monte Carlo 20,000 resamples), *p*_raw = 0.003, *p*_Holm = 0.006, and the achievement-path indirect effect was ab_2_ = 0.064 (95% CI [0.025, 0.107]), *p*_raw = 0.005, *p*_Holm = 0.006. Both 95% confidence intervals excluded zero, and H3 was therefore supported on both the motivation and achievement paths.

**Robustness.** In the LLM-rated secondary outcome model, the achievement-path indirect effect was 0.058 (95% CI [0.020, 0.099]), differing by 0.006 from the primary expert-rated model and remaining significant. The MAI 12-item total-score mediation yielded directionally consistent estimates (motivation ab = 0.076; achievement ab = 0.061, both CIs excluding zero). The Y baseline-proxy check (without the T0 pretest) yielded an achievement-path indirect effect of 0.071 (95% CI [0.030, 0.117]) with 84% CI overlap, above but at the lower end of the prespecified 80% threshold. The within-intervention comparison (T0 → T1 → T2) yielded inflated estimates (0.104 motivation; 0.082 achievement) relative to the T3 model, consistent with the methods-stage expectation (Section 5.2; full results in Supplementary Table S4).

### 4.6. H4 First-Stage Moderated Mediation

H4 was tested on the Arm A and Arm B subsample (*n* = 273; G = 12 classes) to examine the moderating role of prior AI credibility belief on the A versus B indirect effect. The X × W interaction predicting T1 metacognitive regulation was significant (*β* = 0.171, cluster wild bootstrap SE = 0.082, *p* = 0.037), indicating that the a-path effect of A versus B varied with the level of W. Subsample path coefficients were as follows: at W = 0, the a path was 0.043 (95% CI [−0.034, 0.121]; consistent with the H2a main effect); the b_1_ path from T1 metacognition to T3 motivation was 0.357 (95% CI [0.221, 0.491]); and the b_2_ path from T1 metacognition to T3 achievement was 0.263 (95% CI [0.142, 0.382]).

Conditional indirect effects at three levels of W (W = M − 1 SD, M, and M + 1 SD) are summarized in Table 7. At high prior AI credibility belief (W = +1 SD), the conditional indirect effect of A versus B on T3 motivation through T1 metacognitive regulation was 0.077 (95% CI [0.014, 0.144]), and on T3 achievement was 0.057 (95% CI [0.001, 0.115]); both excluded zero, with the achievement effect lying just inside the threshold. At average belief (W = M), the conditional indirect effects were 0.015 for motivation (95% CI [−0.029, 0.061]) and 0.011 for achievement (95% CI [−0.027, 0.052]); both 95% CIs crossed zero. At low belief (W = −1 SD), the conditional indirect effects were −0.046 for motivation (95% CI [−0.114, 0.020]) and −0.034 for achievement (95% CI [−0.097, 0.027]); both trended negative but did not reach significance.

The index of moderated mediation was 0.061 for the motivation path (95% CI [0.013, 0.114]; *p*_raw = 0.017) and 0.045 for the achievement path (95% CI [−0.002, 0.094]; *p*_raw = 0.054). After Holm correction (*k* = 2), the motivation index attained *p*_Holm = 0.034, marginally supported, while the achievement index attained *p*_Holm = 0.054 and did not cross the conventional threshold. H4 is therefore partially supported: the moderated mediation pattern receives marginal evidence on the motivation outcome only, with the achievement-pathway index trending in the predicted direction but failing to reach significance.

**Hayes Model 8 robustness.** In the extended model with W also moderating the X → Y direct paths, the X × W → T3 motivation direct effect was *β* = 0.041 (95% CI [−0.082, 0.168]; *p* = 0.519), and the X × W → T3 achievement direct effect was *β* = 0.027 (95% CI [−0.094, 0.151]; *p* = 0.668). Neither direct moderation path was significant, supporting the methods-stage theoretical assumption that prior trust principally moderates the metacognitive pathway rather than direct pathways. The Model 7 specification for H4 is therefore retained.

The H4 results provide a partial heterogeneity-based account of the non-significant H2a average effect, on the motivation outcome only. The metacognitive-activation effect of AI feedback relative to human feedback exhibited identifiable, opposing contributions across subgroups in the predicted direction, with high-belief learners benefiting and low-belief learners trending negative, though not reaching significance, so that the average motivation-pathway effect was offset toward zero. For the achievement outcome, the same directional pattern was observed. However, the moderation index did not reach significance after Holm correction, so the heterogeneity account is not statistically supported for this outcome despite the directional consistency. This partial pattern is consistent with theoretical predictions in the technology-acceptance literature regarding prior trust as a moderator of technology utility (Komiak & Benbasat, 2006; Lankton et al., 2015), with the caveat that statistical support is restricted to one of the two primary outcomes. We emphasize the appropriate framing of these findings: with the G = 12 cluster constraint, moderate power (~0.60–0.65), the motivation index reaching only the margin of significance after Holm correction (*p*_Holm = 0.034), and the achievement index not reaching significance (*p*_Holm = 0.054), the H4 results should be interpreted as partially supported and exploratory, with replication in larger cluster samples essential—both for replicating the motivation finding and for resolving whether the achievement-pathway null reflects insufficient power or genuine absence of moderation.

### 4.7. Section Summary

Gamification produced an identifiable effect on metacognitive regulation in color education (H1, *d* = 0.34, *p*_Holm = 0.015), below the meta-analytic baseline and below the *d* = 0.50 a priori target but directionally consistent. AI and dose-matched human process feedback did not differ on average (H2a, *d* = 0.07; underpowered, equivalence not inferable). Process feedback yielded a marginal overall increment over gamification alone (H2b, *d* = 0.24, *p*_Holm = 0.048, one of three sensitivity schemes attenuating to non-significance). Metacognitive regulation served as a longitudinal mediator (H3, both 95% CIs excluded zero), with the autoregressive path equal across arms—the intervention shifted M’s level rather than its stability. Prior AI credibility belief moderated the indirect effect of A vs. B through T1 metacognitive regulation only on motivation (H4 motivation, *p*_Holm = 0.034); the achievement-pathway index trended in the predicted direction but did not reach significance (H4 achievement, *p*_Holm = 0.054). High-belief learners benefited on both outcomes (+0.077 motivation, +0.057 achievement); low-belief learners trended negatively on both (−0.046, −0.034) but did not reach significance.

## 5. Discussion

### 5.1. Interpretation of the Principal Findings

**H1 in meta-analytic context.** The gamification effect on metacognitive regulation (*d* = 0.34, *p*_Holm = 0.015) is meaningfully smaller than the meta-analytic composite (*g* = 0.78; Zeng et al., 2024) and falls short of the a priori target of *d* = 0.50, though directionally consistent. Two non-mutually-exclusive interpretations apply: aesthetic subjectivity lengthens the transmission chain from points-based incentives to work-quality outcomes, lowering the ceiling for gamification effects; and the design-undergraduate sample with high baseline AI-tool use (5.2/week) reduces the marginal novelty of gamification mechanics. The result still replicates the SDT–SRL integration framework and extends its applicable range to subjective aesthetic domains, while signaling that future aesthetic-education power calculations should assume smaller effects.

**Joint interpretation of H2a and H4.** H2a yielded a small effect in either direction (Δ = 0.05, 95% CI [−0.13, 0.23] crossing zero). The H4 moderated mediation result partially reframes this null on the motivation outcome only: the average non-significance does not indicate AI–human equivalence but rather heterogeneity, with opposing subgroup contributions whose offset attenuates the average effect toward zero. For the achievement outcome, the same directional pattern was observed but did not reach Holm-corrected significance, so the heterogeneity account holds for only one of the two primary outcomes.

The H4 conditional-effect pattern carries theoretical and practical implications. Theoretically, the pattern is consistent with the technology-trust literature: high-credibility learners integrated AI feedback into metacognitive monitoring (conditional indirect effects: +0.077 for motivation and +0.057 for achievement), whereas low-credibility learners showed defensive skepticism, with non-significant negative trends (−0.046 and −0.034). In practice, differences in sample prior-trust distributions may partially account for inconsistent average effects in prior AI-in-education studies, at least for the motivation outcome. The evidence accordingly does not support any claim that AI feedback is superior to human feedback; the two sources were statistically indistinguishable on average within the available power, and at the conditional level, AI feedback operated, at most, as a trust-dependent complement whose benefit was confined to higher-trust learners and, among the two primary outcomes, to motivation.

The H4 findings’ limitations must be candidly acknowledged: with the G = 12 cluster constraint, moderate power (~0.60–0.65), motivation-path index reaching only marginal significance (*p*_Holm = 0.034), and achievement-path index not significant (*p*_Holm = 0.054), H4 is a partially supported exploratory contribution requiring confirmation in larger-cluster replications.

**Marginal H2b support.** H2b (*d* = 0.24, *p*_Holm = 0.048) provides marginal support for the overall process-feedback increment over gamification alone, but “marginal” applies candidly: *p*_Holm sits essentially at the 0.05 threshold, the effect size is small, design power was ~0.68, and one of three sensitivity schemes did not retain significance. The overall value of process feedback receives marginal, specification-sensitive support; AI specificity is significant only at the conditional level on the motivation outcome (high prior AI credibility belief). This pattern qualifies the AI-feedback increments reported by the predominantly two-arm prior literature (Banihashem et al., 2022; Cavalcanti et al., 2021), since the active-control contrast adopted here isolates the AI-specific component and finds it small on average and conditional at the individual level, suggesting that part of the previously reported AI advantage may reflect the general value of adding process feedback rather than an AI-specific contribution.

### 5.2. Methodological Gain from the Longitudinal Mediation Pathway

Both H3 paths supported longitudinal mediation (both 95% CIs excluded zero), and the cross-arm equality of the autoregressive path was retained—the intervention shifted the level of metacognitive regulation rather than its stability. Instructors can therefore elevate students’ regulatory capacity through structured feedback and gamification without compromising the stability of the regulatory system.

The core methodological gain is placing T3 four weeks after intervention cessation. Fitting the same mediation structure to T2 (Supplementary Table S4) yielded indirect effects of 0.104 (motivation) and 0.082 (achievement)—both higher than the T3 estimates of 0.083 and 0.064. This does not imply that T3 is weaker; rather, T0 → T1 → T2 measures Y while X is still being administered, capturing within-intervention dynamic correlation that does not satisfy the longitudinal mediation identification conditions (Cole & Maxwell, 2003; Maxwell et al., 2011). By placing Y after X has ceased, T0 → T1 → T3 yields smaller but causally cleaner indirect-effect estimates—a concrete methodological reference for future mediation studies in aesthetic education.

### 5.3. Theoretical and Methodological Contributions

The theoretical contribution of the study is the extension of the SDT–SRL integration framework (Ryan & Deci, 2020; Zimmerman, 2008) to the AI-by-gamification context in color education, with three complementary mechanisms identified empirically as candidate transmission pathways within the framework: an independent gamification effect on metacognitive regulation (H1, supported with effect size below the a priori target); an overall process-feedback increment (H2b, marginally supported and specification-sensitive); and conditional heterogeneity in the AI–human contrast as a function of prior trust (H4, partially supported on the motivation pathway only, exploratory in nature, and pending confirmation on the achievement pathway).

The methodological contribution comprises three layers, complemented by one empirical contribution. The first methodological layer: the study is the first to separate AI formative-feedback and human teaching assistant process-feedback contributions in color education through an active-control design with feedback dose strictly matched on four dimensions. The second: prior AI credibility belief was specified a priori as a first-stage moderator on theoretical grounds (Komiak & Benbasat, 2006; Lankton et al., 2015), extending average-effect inference to conditional-effect identification. The third: the combination of four-wave cluster randomization, post-intervention follow-up, cross-arm teacher allocation, and four-dimensional dose matching offers a reusable template. The empirical contribution is to fill a gap in AI interventions in the highly subjective aesthetic domain of color education.

### 5.4. Practical Implications

The findings tentatively support, pending replication, a multi-component framework for hybrid intervention in color education in which gamification mechanics, process feedback, human-expert terminal grading, and prior-trust-aware delivery may complement one another. Gamification mechanics sustain motivational activation; process feedback (whether from AI or human sources) activates metacognitive regulation, with the magnitude of this contribution sensitive to specification choices; human experts handle terminal grading to ensure the contextual sensitivity of aesthetic judgment; and prior-trust screening on the motivation outcome may help identify learners less likely to benefit from AI feedback, although this implication is not yet supported on achievement outcomes and the underlying mechanism requires replication.

The 0.31-unit advantage in perceived feedback continuity, favoring AI over human teaching assistants (Section 4.2), is most parsimoniously explained as a byproduct of AI-generated feedback’s formal consistency (uniform structuring, fixed templating, absence of tone variation), rather than dose stability—given that dose was matched on frequency, dimension count, word count, and timing. Whether this perceptual difference contributes value beyond matched dose, and whether learners interpret it as a quality cue or a mechanical artifact, are open questions.

AI tools in aesthetic education are best positioned as process-feedback generators rather than terminal graders, with utility that may depend on learners’ prior trust. The high-belief conditional indirect effect for motivation (+0.077, 95% CI [0.014, 0.144]) and for achievement (+0.057, 95% CI [0.001, 0.115], lower bound just inside the threshold), combined with the negative trends at low belief (−0.046 and −0.034, neither significant), provides preliminary support—within the limits of an exploratory analysis on *n* = 273 and G = 12—for the claim that AI feedback may serve as a cost-effective complement to human teaching assistants in resource-constrained color-education courses, particularly for learners at or above the median of prior AI credibility belief. We emphasize the distinction between two statistical quantities: the conditional indirect effect at a specific level of W reflects a point inference. In contrast, the moderated mediation index reflects the inference of moderation itself. The achievement-pathway conditional effect at W = +1 SD reached significance at the single-point level, but the corresponding moderation index did not reach Holm-corrected significance (*p*_Holm = 0.054); these two quantities address distinct inferential questions, and only the latter constitutes support for the H4 hypothesis on the achievement outcome. For low-trust learners, the absence of statistically significant negative effects does not establish safety; the negative trends warrant caution, and resources may reasonably be allocated preferentially to human feedback or to preliminary AI-trust-building interventions. We discourage the prescriptive deployment of trust-screening protocols in AI-supported color education courses based solely on the present study.

A final design feature qualifies the preceding interpretation: whether students were aware of the source of their process feedback. In the present trial, students in Arms A and B were informed whether their feedback was generated by an AI system or written by a human teaching assistant (Section 3.2), and this disclosure has two consequences for how the results should be interpreted. First, the AI-versus-human contrast (H2a) and its conditional form (H4) reflect not only any difference in the feedback itself but also the expectancy that source knowledge introduces: a learner who knows that commentary is machine-generated may weigh identical commentary differently from one who attributes it to a human, independent of its content. The small average H2a effect, together with the marginally higher perceived feedback continuity in Arm A (Δ = 0.31, *p* = 0.032; Section 4.2), is therefore most cautiously read as the joint product of feedback content and source-driven expectancy rather than of content alone. Second, disclosure is precisely what renders the H4 moderation mechanistically coherent: prior AI credibility beliefs can shape the uptake of AI feedback only if learners recognize it as AI-generated, so the trust-dependent pattern reported here presupposes source awareness rather than standing apart from it. These considerations reinforce the cautious framing adopted throughout. The conditional AI benefit observed here operated within a context of known source, and a fully blinded replication—in which learners receive structurally identical feedback without knowing its origin—would be required to separate the contribution of feedback content from that of source expectancy. This boundary is noted alongside the cluster-count and power limitations of Section 5.5.

### 5.5. Limitations and Future Directions

Several limitations should be acknowledged, the most consequential being the small number of clusters and the limited statistical power of the AI-versus-human comparison and the moderated-mediation analyses. Cross-arm allocation of eight teachers addresses but cannot fully eliminate residual teacher characteristics under cluster-robust SEs; multi-site replication should expand teacher samples to ≥16. The 24-cluster count falls below the multilevel SEM threshold of 30+, making wild-cluster bootstrap a second-best inference method at this scale. H2a power (~0.50) prevents the inference of equivalence between AI and human feedback. H2b (*p*_Holm = 0.048) was marginal and specification-sensitive (one of three sensitivity schemes attenuated to non-significance). H4 on the A/B subsample (*n* = 273; G = 12) had moderate power (~0.60); only the motivation-pathway index reached marginal significance, while the achievement-pathway index did not. Replication in larger cluster samples is required before either of the H4 findings can be elevated to confirmatory status. The Y baseline-proxy yielded 84% CI overlap, at the lower end of the prespecified 80% threshold.

The 18-week window is short; long-term effects beyond six months were not assessed. The single-institution sample limits cross-cultural and cross-disciplinary generalizability, and the aesthetic character of color education imposes a further boundary on transfer: because evaluation in this domain carries inherent subjectivity, the metacognitive-activation pattern observed here may not extend in the same form to objective or technical subjects, in which external evaluation is less uncertain and learners rely less heavily on internal regulation, so that the relative contributions of gamification, process feedback, and prior trust could differ. AI model version drift was addressed through an enterprise SLA fixing ERNIE-4.0-8K-0613 for the 14-week window only. Common-method bias did not pose a substantive threat per Harman’s test and the marker-variable method.

The MAI cognitive-regulation subscale AVE of 0.48 is slightly below the 0.50 threshold, and its discriminant pairing with the MSLQ self-efficacy scale yielded HTMT = 0.86, between the strict (0.85) and lenient (0.90) cutoffs of Henseler et al. (2015); the simultaneous appearance of these two values means that discriminant validity at this scale-pair is not strongly supported. The H3 motivation-path indirect effect should therefore be interpreted as partially confounded with metacognition–self-efficacy construct overlap, and the MAI cognitive-regulation subscale’s psychometric properties require further refinement in subsequent studies—possibly through item revision, expansion to the full MAI rather than the short form, or use of a different metacognition instrument with cleaner discriminant structure relative to motivation constructs. The 4-item adaptation of the Lankton et al. (2015) functionality and reliability subscales passed expert content-validity review (CVI = 0.83) but inevitably sacrifices construct coverage relative to the original full subscales; future studies in larger samples should employ the full subscales without item reduction.

Future directions include cross-disciplinary and cross-cultural replication in other aesthetic education domains (music, dance, writing); long-term follow-up at six and twelve months; an RCT design for AI-trust-building interventions, with reassessment of AI-feedback effects following a preliminary trust-building intervention for high- and low-trust learners; a complete 2 × 2 factorial design (AI vs. no AI × gamification vs. no gamification) to separate the interaction of the two intervention components; stratified analyses by T0 metacognitive baseline to identify which learners benefit most; and most urgently, larger-cluster replication powered to resolve whether the H4 achievement-pathway null reflects insufficient power or genuine outcome-specific bounds on the moderation pattern.

## 6. Conclusions

This four-arm × four-wave cluster RCT with cross-arm teacher allocation, post-intervention follow-up, and an a priori moderated mediation framework separated AI formative feedback and human teaching assistant process feedback contributions in color education for the first time, while extending average-effect inference to conditional-effect identification through prior AI credibility belief. Strict four-dimensional dose matching, T3 measurement four weeks after intervention cessation, and cluster wild bootstrap pushed design-level causal-identification rigor to the practical upper bound for studies of this scale.

Gamification produced an identifiable effect on metacognitive regulation (H1, *d* = 0.34), below the meta-analytic composite (*g* = 0.78) but directionally consistent—reflecting the ceiling-pressure of aesthetic subjectivity. AI and dose-matched human feedback did not differ on average (H2a, *d* = 0.07; equivalence not inferable). H2b (*d* = 0.24, *p*_Holm = 0.048) was marginal and specification-sensitive. Metacognitive regulation mediated the intervention’s effect on T3 motivation and achievement (H3; both 95% CIs excluded zero). Prior AI credibility belief moderated this indirect effect only on motivation as exploratory evidence (H4 motivation, *p*_Holm = 0.034), with high-belief learners benefiting and low-belief learners trending negative; the achievement-outcome moderation index did not reach significance (H4 achievement, *p*_Holm = 0.054).

The study fills an empirical gap in AI interventions in color education; addresses the AI-specificity question through active-control dose matching; extends average-effect to conditional-effect inference with prior trust as a candidate source of motivation-outcome heterogeneity; provides genuine longitudinal mediation evidence with Y measured after X cessation; and offers a multi-component framework—gamification, process feedback, human-expert terminal grading, prior-trust-aware delivery—as a tentative organizing structure for AI-supported aesthetic education. The framework’s prescriptive value depends on replications that confirm H4 across both outcomes and resolve the specification sensitivity of H2b.

## Figures and Tables

**Table 1:** Hypotheses, analytic strategies, and power targets.

Hyp.	Statement	Contrast/Path	Outcome (T3)	Method	Direction/Effect	Family
H1	Gamification surpasses traditional instruction in metacognitive regulation.	C vs. D	MAI cognitive regulation subscale (6 items)	Cluster-robust ANCOVA	Directional prediction (C > D); two-sided test; *d* = 0.50	Family 1 (k = 3)
H2a	AI feedback differs from human TA feedback (no direction predicted)	A vs. B	Same as H1	Cluster-robust ANCOVA	Two-sided; *d* = 0.40	Family 1 (k = 3)
H2b	Pooled process feedback surpasses gamification alone	A + B vs. C	Same as H1	Cluster-robust ANCOVA	Directional prediction (A + B > C); two-sided test; *d* = 0.30	Family 1 (k = 3)
H3-Mot	T1 metacognition mediates intervention → T3 motivation	T0 → T1 → T3	MSLQ composite (15 items)	Autoregressive mediation; Mplus; Monte Carlo CI 20,000	Two-sided; ab ≈ 0.10	Family 2 (k = 2)
H3-Ach	T1 metacognition mediates intervention → T3 achievement	T0 → T1 → T3	Color-work achievement (3 expert raters, 5-dim. rubric)	Same as above	Two-sided; ab ≈ 0.10	Family 2 (k = 2)
H4	T0 AI credibility belief moderates the A vs. B indirect effect through T1 metacognition.	(A vs. B) × W → T1 M → T3 Y	Same as H3 paths	Hayes Model 7; cluster wild bootstrap CI 5000	Two-sided; index ≈ 0.05	Family 3 (k = 2)

*Note.* Covariates throughout include T0 same-name variables, demographic variables (sex, year of study, AI-tool use frequency, digital literacy, prior color-course experience), and teacher dummies. Three independent Holm (1979) corrections are applied to Family 1 (k = 3), Family 2 (k = 2), and Family 3 (k = 2); pooled correction across families would be excessively conservative. Primary analyses use FIML on N = 540 (A = 140, B = 133, C = 138, D = 129) based on T0 completion; complete-case sensitivity analyses use N = 456. MAI = Metacognitive Awareness Inventory; MSLQ = Motivated Strategies for Learning Questionnaire.

**Table 2:** CONSORT-extension flow diagram for the cluster randomized trial.

Stage	Arm A	Arm B	Arm C	Arm D	Total
Classes assessed for eligibility	9	7	8	7	31
Classes excluded	3	1	2	1	7
Reasons for exclusion	Maternity 1; transfer 1; class size 1	Reassignment 1	Transfer 1; timetable 1	Maternity 1	7
Classes randomized	6	6	6	6	24
Students invited at T0 (registered before unmasking)	142	135	139	133	549
Missing at T0 (illness/refusal)	2	2	1	4	9
Completed T0 baseline	140	133	138	129	540
Completed T1 (week 9)	134	129	134	122	519
Completed T2 (week 14)	128	124	130	115	497
Completed T3 (week 18)	118	114	121	103	456
Cumulative T3 attrition (%)	22 (15.7%)	19 (14.3%)	17 (12.3%)	26 (20.2%)	84 (15.6%)
Primary analysis sample (FIML)	140	133	138	129	540
Complete-case sample	118	114	121	103	456

*Note.* Cluster-level randomization was completed before T0; T0 invitations by assigned class served only as administrative records, and students did not yet know their class’s assignment when completing T0. The asymmetric distribution of candidate classes across arms (9/7/8/7) reflects the actual distribution of available courses across timetable slots within the design school. Arm A T2 attrition (8.6%) was slightly higher than Arm B (6.8%), a counter-expected pattern that may reflect mid-intervention novelty decay of AI-generated feedback (see Section 5.1). Arm D had the highest cumulative attrition (20.2%), consistent with the well-known pattern of accelerated motivational attrition in control conditions. Little’s MCAR test confirmed that missing data were consistent with a completely-at-random pattern, and FIML was used for the primary analyses. CONSORT = Consolidated Standards of Reporting Trials; FIML = full-information maximum likelihood; MCAR = missing completely at random.

**Table 3:** Comparison of intervention components across the four arms.

Component	Arm A	Arm B	Arm C	Arm D
Feedback frequency	Weekly	Weekly	—	End of term only
Feedback source	ERNIE 4.0	Trained human TA	—	—
Number of feedback dimensions	5 (fixed)	5 (matched to A)	—	—
Feedback word-count median (IQR)	308 (271–352)	296 (246–338)	—	—
Dimension-coverage completeness	96.7%	93.4%	—	—
Feedback timing (submission to feedback, median h)	18	22	—	—
Gamification mechanics (points/unlock/dashboard/badges/archive)	Yes	Yes	Yes	No
Teacher cross-arm distribution	See Supplementary S2	See Supplementary S2	See Supplementary S2	See Supplementary S2
Number of teaching assistants	—	6 (one per class)	—	—

*Note.* Arms A and B were strictly matched on the four dose dimensions; the only difference was the feedback source. The slightly higher dimension-coverage completeness of AI feedback (96.7% vs. 93.4%) reflects the structural stability of large-language-model output under temperature = 0; the gap from 100% reflects occasional JSON parsing failures (~3.3%) that were resolved through manual back-up review. The interquartile range overlap of feedback word counts was 86%, and the keyword Jaccard overlap was 0.78, indicating dose matching at both the textual-volume and content levels. TA = teaching assistant; IQR = interquartile range.

**Table 4:** A priori power analysis across sample sizes and ICC scenarios.

Hyp.	Test	Target Effect	N = 540, ICC = 0.05	N = 540, ICC = 0.10	N = 540, ICC = 0.15	N = 456, ICC = 0.10	Power Note
H1	C vs. D (directional prediction; two-sided)	*d* = 0.50	0.81	0.73	0.66	0.67	Approaches 0.80 when ICC ≤ 0.10
H2a	A vs. B (two-sided)	*d* = 0.40	0.57	0.49	0.43	0.43	Underpowered; non-significance does not imply equivalence.
H2b	A + B vs. C (directional prediction; two-sided)	*d* = 0.30	0.74	0.68	0.62	0.62	Slightly below 0.80
H3 Mot.	T0 → T1 → T3 indirect	ab ≈ 0.10	0.85	0.78	0.71	0.72	Approaches 0.80
H3 Ach.	T0 → T1 → T3 indirect	ab ≈ 0.10	0.82	0.75	0.69	0.69	Approaches 0.80
H4 Mot.	(A vs. B) × W → M → Y	index ≈ 0.05	0.71	0.64	0.58	0.58	Moderate; G = 12 inflates uncertainty
H4 Ach.	(A vs. B) × W → M → Y	index ≈ 0.05	0.68	0.61	0.55	0.55	Moderate; further pressure from Y construct mismatch

*Note.* The H1 target effect of *d* = 0.50 was set as a conservative lower bound rather than the meta-analytic composite *g* = 0.78, on the assumption that aesthetic subjectivity in color education would attenuate gamification effects. Under the baseline ICC = 0.10, only the two H3 paths approached the 0.80 threshold; H1 and H2b were slightly below; H2a was substantially underpowered (~0.50) across all scenarios; and the two H4 paths reached only moderate power (~0.60–0.65). The G = 12 cluster constraint implies that H4 statistical inference is associated with substantial uncertainty across all scenarios, and H4 results should be interpreted as exploratory rather than confirmatory. Power estimates were obtained with Optimal Design 4.0, and Mplus Monte Carlo simulation (10,000 resamples, with cluster-nesting correction) under two-sided testing for all hypotheses; for H1 and H2b, the directional theoretical predictions enter the interpretation of the predicted sign of Δ_adj but not the statistical decision rule, consistent with the conservative analytic choice described in Section 3.4. ICC = intracluster correlation.

**Table 5:** Sample characteristics, baseline equivalence, and intraclass correlations.

Variable	Arm A (*n* = 140)	Arm B (*n* = 133)	Arm C (*n* = 138)	Arm D (*n* = 129)	F	*p*	ICC [95% CI]
Sex (% female)	64.3	67.7	65.2	62.0	0.61	0.612	0.012 [0.000, 0.071]
Year (% Y2/Y3)	58.6/41.4	60.2/39.8	56.5/43.5	59.7/40.3	0.34	0.793	—
AI-tool use (per week)	5.6 ± 3.2	4.8 ± 2.7	5.4 ± 3.1	5.1 ± 3.0	1.84	0.139	0.027 [0.000, 0.094]
Digital literacy self-rating (1–5)	3.78 ± 0.61	3.61 ± 0.66	3.74 ± 0.59	3.69 ± 0.64	1.96	0.119	0.034 [0.000, 0.108]
Prior color-course experience (1–5)	2.91 ± 0.88	2.85 ± 0.91	2.87 ± 0.94	2.64 ± 0.92	2.21	0.087	0.054 [0.011, 0.131]
T0 metacognitive regulation (MAI)	3.44 ± 0.68	3.36 ± 0.72	3.48 ± 0.65	3.39 ± 0.69	0.84	0.475	0.083 [0.041, 0.142]
T0 metacognitive knowledge (MAI)	3.58 ± 0.71	3.49 ± 0.73	3.61 ± 0.69	3.51 ± 0.74	0.92	0.430	0.072 [0.034, 0.128]
T0 learning motivation (MSLQ)	3.69 ± 0.74	3.66 ± 0.71	3.81 ± 0.69	3.62 ± 0.73	2.74	0.043	0.064 [0.027, 0.117]
T0 color-knowledge pretest (%)	62.7 ± 11.4	60.8 ± 12.2	63.4 ± 11.6	61.9 ± 12.0	1.13	0.337	0.058 [0.022, 0.108]
T0 AI credibility belief (1–5)	3.51 ± 0.78	3.39 ± 0.81	3.46 ± 0.76	3.42 ± 0.78	0.61	0.609	0.041 [0.000, 0.114]

*Note.* F and *p* values are from cluster-robust ANCOVAs with class as cluster and teacher dummies as covariates. Two marginal differences—prior color-course experience (*p* = 0.087) and T0 learning motivation (*p* = 0.043)—were addressed through covariate adjustment in the primary analyses, with unadjusted models reported as sensitivity references. The *p*-value distribution is consistent with the genuine sampling distribution under restricted randomization without post hoc baseline balancing (1–2 marginal differences expected among 10 variables). The T0 metacognitive regulation ICC (0.083) was slightly below the method’s baseline of 0.10 and was used in the cluster-robust calibration of the primary analyses. MAI = Metacognitive Awareness Inventory; MSLQ = Motivated Strategies for Learning Questionnaire.

**Table 6:** Manipulation-check items at T1 (cluster-robust ANCOVA).

Item	Arm A	Arm B	Arm C	Arm D	F	*p*
The course provided me with clear feedback on my progress.	4.21 ± 0.74	4.09 ± 0.81	3.74 ± 0.86	2.83 ± 0.97	47.32	<0.001
The course gave me specific, actionable suggestions.	4.18 ± 0.79	4.13 ± 0.83	3.41 ± 0.89	2.65 ± 1.02	62.18	<0.001
I experienced the feedback as immediate and continuous.	4.27 ± 0.71	3.96 ± 0.85	3.62 ± 0.88	2.78 ± 1.04	51.46	<0.001

*Note.* Means ± standard deviations on a 5-point Likert scale, controlling for T0 covariates. The A vs. B contrast (12 classes) used Bell–McCaffrey corrected degrees of freedom, t(10). For the first two items, A vs. B differences were non-significant (Δ = 0.12, *t*(10) = 0.84, *p* = 0.421; Δ = 0.05, *t*(10) = 0.36, *p* = 0.726), consistent with the dose-matching design. Arm A scored marginally higher than Arm B on perceived feedback continuity (Δ = 0.31, *t*(10) = 2.49, *p* = 0.032). Arm C scored significantly lower than Arms A and B but significantly higher than Arm D, confirming the intermediate position of progress feedback delivered by gamification. ANCOVA = analysis of covariance.

**Table 7:** Main effects (H1, H2a, H2b), longitudinal mediation (H3), and moderated mediation (H4).

Test	Contrast/Path	Estimate	95% CI	*d*/*β*	*p*_raw	*p*_Holm	Conclusion
*Main effects (H1, H2a, H2b cluster-robust ANCOVA)*							
H1	C vs. D (Δ_adj)	0.23	[0.10, 0.36]	*d* = 0.34	0.005	0.015	Supported
H2a	A vs. B (Δ_adj, two-sided)	0.05	[−0.13, 0.23]	*d* = 0.07	0.555	0.555	No avg. difference (underpowered)
H2b	A + B vs. C (Δ_adj, two-sided)	0.17	[0.02, 0.32]	*d* = 0.24	0.024	0.048	Marginal; specification-sensitive
Expl.	A vs. C (Δ_adj)	0.19	[0.04, 0.34]	*d* = 0.27	0.017	—	Descriptive
Expl.	A vs. D (Δ_adj)	0.42	[0.25, 0.59]	*d* = 0.62	<0.001	—	Descriptive
*H3 longitudinal mediation (T0 → T1 → T3 autoregressive mediation; full sample, N = 540)*							
H3	T0 → T1 autoregression	0.582	[0.521, 0.638]	*β*	<0.001	—	Equality retained
H3	X → T1 metacognition (a path)	0.224	[0.128, 0.319]	*β*	<0.001	—	M is affected by X
H3 Mot.	T1 metacog. → T3 motivation (b_1_)	0.371	[0.265, 0.476]	*β*	<0.001	—	—
H3 Mot.	Indirect effect ab_1_	0.083	[0.039, 0.135]	*β*	0.003	0.006	Supported
H3 Mot.	X → T3 motivation (c′_1_ direct)	0.118	[0.029, 0.205]	*β*	0.009	—	Direct effect significant
H3 Ach.	T1 metacog. → T3 achievement (b_2_)	0.286	[0.176, 0.394]	*β*	<0.001	—	—
H3 Ach.	Indirect effect ab_2_	0.064	[0.025, 0.107]	*β*	0.005	0.006	Supported
H3 Ach.	X → T3 achievement (c′_2_ direct)	0.091	[0.008, 0.176]	*β*	0.032	—	Direct effect significant
*H4 first-stage moderated mediation (A vs. B subsample, n = 273, G = 12; W = T0 AI credibility belief)*							
H4	a path (X main effect at W = 0)	0.043	[−0.034, 0.121]	*β*	0.278	—	n.s. at W = 0
H4	a_3_: X × W → T1 M interaction	0.171	[0.018, 0.321]	*β*	0.037	—	Interaction significant
H4 sub.	b_1_: T1 M → T3 motivation	0.357	[0.221, 0.491]	*β*	<0.001	—	—
H4 sub.	b_2_: T1 M → T3 achievement	0.263	[0.142, 0.382]	*β*	<0.001	—	—
H4 Mot.	Conditional indirect at W = +1 SD	0.077	[0.014, 0.144]	*β*	0.019	—	Significantly positive
H4 Mot.	Conditional indirect at W = M	0.015	[−0.029, 0.061]	*β*	0.515	—	n.s.
H4 Mot.	Conditional indirect at W = −1 SD	−0.046	[−0.114, 0.020]	*β*	0.157	—	Trend negative; n.s.
H4 Mot.	Index of moderated mediation	0.061	[0.013, 0.114]	*β*	0.017	0.034	Marginally supported
H4 Ach.	Conditional indirect at W = +1 SD	0.057	[0.001, 0.115]	*β*	0.047	—	Sig. positive (margin)
H4 Ach.	Conditional indirect at W = M	0.011	[−0.027, 0.052]	*β*	0.587	—	n.s.
H4 Ach.	Conditional indirect at W = −1 SD	−0.034	[−0.097, 0.027]	*β*	0.263	—	Trend negative; n.s.
H4 Ach.	Index of moderated mediation	0.045	[−0.002, 0.094]	*β*	0.054	0.054	Not supported (trend)

*Note.* Adjusted means (Δ_adj) control for T0 metacognition, T0 learning motivation, T0 color-knowledge pretest, T0 AI credibility belief, demographic controls, and teacher dummies. Holm correction is applied within three families: H1/H2a/H2b (k = 3); H3 paths (k = 2); and H4-moderated mediation indices (k = 2). H3 indirect effects use Monte Carlo (20,000 resamples); H4 indirect effects use cluster wild bootstrap (5000 resamples; 6-point Webb distribution) to address the G = 12 cluster constraint. Bell–McCaffrey-corrected degrees of freedom were used: H1 t(10), H2a t(10), and H2b t(16). *β* = standardized regression coefficient; n.s. = not significant.

## Data Availability

The original contributions presented in this study are included in the article/Supplementary Material. Further inquiries can be directed to the corresponding author.

## Supplementary Materials

The following supporting information can be downloaded at: https://www.mdpi.com/article/10.3390/bs16071247/s1, Supplementary S0: Eight-week pilot study (procedural-pipeline verification); Supplementary S1: Sensitivity analyses excluding classes affected by the three unanticipated implementation events; Supplementary S2: Full 8 × 24 teacher cross-arm allocation matrix and arm-specific competence-assessment outcomes; Supplementary S3: Illustrative feedback examples for Arm A (AI) and Arm B (human teaching assistant); Supplementary S4: T0 → T1 → T2 within-intervention mediation; Supplementary S5: Prior AI credibility belief 4-item scale; Table S1: Sensitivity-analysis effect sizes across schemes excluding affected classes; Table S2: Teacher × class allocation across four arms; Table S3: Constructed illustrative feedback examples for Arm A and Arm B across the five feedback dimensions; Table S4: T0 → T1 → T2 within-intervention mediation, parallel to Table 7; Table S5: Baseline equivalence sensitivity references and H1/H2a/H2b sensitivity-analysis results.

## Abbreviations

The following abbreviations are used in this manuscript:

AI	artificial intelligence
RCT	randomized controlled trial
SRL	self-regulated learning
SDT	self-determination theory
MAI	Metacognitive Awareness Inventory
MSLQ	Motivated Strategies for Learning Questionnaire
ICC	intraclass correlation
FIML	full-information maximum likelihood
MCAR	missing completely at random
CONSORT	Consolidated Standards of Reporting Trials
ANCOVA	analysis of covariance
CFA	confirmatory factor analysis
CFI	comparative fit index
TLI	Tucker–Lewis index
RMSEA	root mean square error of approximation
SRMR	standardized root mean square residual
CR	composite reliability
AVE	average variance extracted
HTMT	heterotrait–monotrait ratio
CI	confidence interval
SE	standard error
IQR	interquartile range
TA	teaching assistant
SLA	service-level agreement
LLM	large language model
API	application programming interface
CVI	content validity index
SEM	structural equation modeling
