# Supplementary material for: AI Versus Human Feedback in Gamified Color Education: A Four-Arm Cluster Randomized Controlled Trial
Source: Behav Sci (Basel). 2026 Jul 22;16(7):1247. doi: 10.3390/bs16071247 (PMC13405382; doi:10.3390/bs16071247)
Supplement: Supplementary file 1 [file behavsci-16-01247-s001.zip › behavsci-4354245-supplementary.pdf]

Supplementary Material

AI-Powered Formative Assessment in Gamified Color Education: A Moderated Mediation Model of Metacognitive Regulation Conditional on Prior AI Credibility Belief, with Effects on Learning Motivation and Achievement Among Design Students in China

Contents

S0. Eight-week pilot study (n = 46, two classes): procedural-pipeline verification

S1. Sensitivity analyses excluding classes affected by the three unanticipated implementation events

S2. Full 8 × 24 teacher cross-arm allocation matrix and arm-specific competence-assessment outcomes

S3. Illustrative feedback examples for Arm A (AI) and Arm B (human teaching assistant) across the five feedback dimensions

S4. T0 → T1 → T2 within-intervention mediation: full results and methodological discussion

S5. Prior AI credibility belief 4-item scale: original 6-item source, 4-item adapted version, item-selection rationale, and Chinese translation

Table S4. T0 → T1 → T2 within-intervention mediation, parallel to Table 7

Table S5. Baseline equivalence sensitivity references and H1/H2a/H2b sensitivity-analysis results across three specification schemes

S0. Eight-week pilot study (n = 46, two classes)

S0.1 Purpose and design

An eight-week pilot study was conducted prior to the main 14-week trial to verify the feasibility of the full procedural pipeline. The pilot was not designed to detect intervention effects (n = 46 is far below the power threshold for any hypothesis in Table 1); its sole purpose was to (a) verify the technical reliability of the ERNIE 4.0 enterprise API under the prescribed parameters, (b) calibrate the human teaching-assistant feedback-writing protocol against the AI dimensional template, (c) confirm that the four-dimensional dose-matching scheme was operationally achievable, and (d) validate the psychometric performance of the 4-item prior AI credibility belief scale (Lankton et al., 2015) in the target population.

The pilot enrolled 46 design undergraduates from two intact classes at the same institution as the main study, in the academic semester immediately preceding the main trial. The two classes were assigned to a pilot Arm A condition (AI formative feedback + gamification; n = 23) and a pilot Arm B condition (human teaching-assistant feedback + gamification; n = 23). Pilot participants did not subsequently enroll in any of the 24 main-trial classes.

S0.2 Pilot results

Procedural pipeline. All eight weekly assignment cycles in both pilot arms completed successfully. Feedback delivery rates were 96.4% (pilot Arm A) and 92.1% (pilot Arm B), within 1.5 percentage points of the rates subsequently observed in the main trial (95.7% and 91.4%). Median feedback-delivery latency was 16 h (pilot Arm A) and 23 h (pilot Arm B), comparable to the main-trial values (18 h and 22 h).

AI system reliability. Across 184 ERNIE 4.0 API calls in pilot Arm A, four (2.2%) returned malformed JSON requiring manual back-up review; the remaining 180 calls returned valid structured output within the prescribed 5-dimension template. Cumulative agreement with three independent color-education experts (ICC[2,k] on the n = 46 pilot sample) was 0.79, above the prespecified 0.75 threshold. Test–retest stability at two weeks on a sub-sample (n = 18) yielded ICC = 0.81.

Dose matching feasibility. In the pilot, AI and human feedback differed by 8 characters in median word count (308 vs. 300), 2.5 percentage points in dimension-coverage rate (96.1% vs. 93.6%), 7 h in median delivery latency, and 0.76 in keyword Jaccard overlap. All four indicators fell within the dose-matching tolerance ranges subsequently used in the main trial. Inter-rater agreement among the three independent color-education experts in scoring the same pilot color works was ICC(2,3) = 0.77.

Prior AI credibility belief scale. The 4-item adapted scale (functionality + reliability subscales, 2 items each; see S5) achieved Cronbach's $\alpha = 0.81$ in the pilot sample. Three independent education-measurement experts conducted a content-validity review yielding a content-validity index (CVI) of 0.83. Item-total

correlations ranged 0.62–0.74. The 4-item version was therefore retained for the main study.

Issues identified and resolutions. Two procedural issues identified in the pilot led to refinements in the main trial: (1) the original 200–500 character feedback word-count range yielded excessive variance, and was tightened to 200–400 characters in the main trial; (2) the AI dimension-coverage rate dropped below 95% on assignments involving complex color symbolism not covered in the system prompt, prompting expansion of the system prompt's five-dimension scoring guidance prior to the main trial.

S1 Sensitivity analyses excluding classes affected by the three unanticipated implementation events

Three unanticipated implementation events occurred during the 14-week main intervention (Methods 3.1, p. 7): (a) one Arm A class experienced a four-day feedback delay in week 11 due to a sports-day timetable adjustment; (b) one Arm B teaching assistant was absent for three days in week 14 due to a family emergency, with backup-delivered feedback; (c) one Arm A class experienced a five-hour AI-system outage in week 7 during scheduled campus-network maintenance, with feedback delivered via backup API endpoint and median response time temporarily elevated from 18 h to 32 h. None of these events constituted a protocol deviation in the predefined sense. Nevertheless, three independent sensitivity-analysis schemes were performed to verify that principal conclusions are not driven by these classes.

S1.1 Specification of the three sensitivity schemes

Scheme 1: Exclude the two Arm A classes from all analyses (classes affected by events (a) and (c)); 25 students excluded from FIML sample; sensitivity-analysis N = 515.

Scheme 2: Exclude the one Arm B class affected by event (b); 21 students excluded; sensitivity-analysis N = 519.

Scheme 3: Exclude all three affected classes simultaneously; 46 students excluded; sensitivity-analysis N = 494.

S1.2 Results

Effect-size estimates for H1, H2a, H2b, H3, and H4 across the three sensitivity schemes are reported in Table S1. Direction and significance of all five hypothesis conclusions were preserved across all three schemes:

• H1 (C vs. D): $d = 0.34$ (primary), 0.32 (Scheme 1), 0.34 (Scheme 2), 0.32 (Scheme 3). All p_Holm < .02.

• H2a (A vs. B): $d = 0.07$ (primary), 0.06 (Scheme 1), 0.08 (Scheme 2), 0.06 (Scheme 3). All p_Holm > .50.

• H2b (A+B vs. C): $d = 0.24$ (primary), 0.22 (Scheme 1), 0.24 (Scheme 2), 0.22 (Scheme 3). H2b p_Holm remained marginal across all schemes (.048 primary → .051 in Scheme 1 → .048 in Scheme 2 → .052 in Scheme 3); the principal H2b conclusion's specification-sensitivity is independent of the three implementation events.

• H3 motivation-path ab_1: 0.083 (primary) → 0.080, 0.084, 0.078 (Schemes 1–3); all CIs excluded zero.

• H3 achievement-path ab_2: 0.064 (primary) → 0.063, 0.064, 0.061 (Schemes 1–3); all CIs excluded zero.

• H4 motivation-path moderation index: 0.061 (primary) → 0.057, 0.062, 0.058 (Schemes 1–3); p_Holm

= .034 → .039, .033, .041 across the three schemes (motivation pathway remains marginally supported in all schemes).

• H4 achievement-path moderation index: 0.045 (primary) → 0.043, 0.045, 0.041 (Schemes 1–3); *p*_Holm = .054 in primary, .062, .054, .069 across the three schemes (achievement pathway remains non-significant in all schemes).

These results indicate that excluding the affected classes does not alter the principal conclusions on any of the five hypotheses, supporting the methods-stage decision to retain these classes in the primary analyses.

S1.3 Table S1

Table S1. *Sensitivity-analysis effect sizes across schemes excluding affected classes*

**Hypothesis**	**Primary (N = 540)**	**Scheme 1 (N = 515)**	**Scheme 2 (N = 519)**	**Scheme 3 (N = 494)**
H1 (d)	0.34**	0.32**	0.34**	0.32**
H2a (d)	0.07	0.06	0.08	0.06
H2b (d)	0.24*	0.22 (margin)	0.24*	0.22 (margin)
H3 Mot. ab_1	0.083***	0.080***	0.084***	0.078***
H3 Ach. ab_2	0.064***	0.063***	0.064***	0.061***
H4 Mot. index	0.061*	0.057*	0.062*	0.058 (margin)
H4 Ach. index	0.045 n.s.	0.043 n.s.	0.045 n.s.	0.041 n.s.

Note. Scheme 1 excludes the two Arm A classes affected by events (a) sports-day delay and (c) network outage; Scheme 2 excludes the Arm B class affected by event (b) teaching-assistant absence; Scheme 3 excludes all three classes simultaneously. p-value markers: * p_Holm < .05; ** p_Holm < .01; *** p_Holm < .001; n.s. = not significant; margin = .045 < p_Holm ≤ .065 borderline.

S2. Full 8 × 24 teacher cross-arm allocation matrix

S2.1 Allocation principles and constraints

The eight participating teachers (T1–T8) were each assigned to three classes across the 14-week intervention. Cross-arm allocation was constrained by two principles to identify teacher main effects separately from treatment main effects: (1) each teacher covered at least two intervention conditions (ensuring teacher × treatment identifiability), and (2) each of the four arms was covered by at least four teachers (avoiding teacher–treatment collinearity).

Under these two constraints, the allocation pattern in which each teacher's three classes were assigned across three different arms—rather than concentrated in one or two arms—was the only configuration that simultaneously satisfied the 24-of-32 cell-coverage requirement and kept each teacher's class load equal at three classes. The pre-randomization enumeration identified 184 feasible allocation schemes; the actual scheme used in the main trial was drawn with intermediate selection probability after the seven excluded classes had been removed.

S2.2 Full 8 × 24 allocation matrix

Table S2. *Teacher × class allocation across four arms*

Teacher	Class 1 (Arm)	Class 2 (Arm)	Class 3 (Arm)	Arms covered	A classes	B classes	C classes	D classes
T1	1 (A)	9 (B)	17 (C)	A, B, C	1	1	1	0
T2	2 (A)	10 (B)	19 (D)	A, B, D	1	1	0	1
T3	3 (A)	13 (C)	20 (D)	A, C, D	1	0	1	1
T4	4 (A)	11 (B)	14 (C)	A, B, C	1	1	1	0
T5	5 (A)	15 (C)	21 (D)	A, C, D	1	0	1	1
T6	6 (A)	12 (B)	22 (D)	A, B, D	1	1	0	1
T7	7 (B)	16 (C)	23 (D)	B, C, D	0	1	1	1
T8	8 (B)	18 (C)	24 (D)	B, C, D	0	1	1	1
Total	—	—	—	—	6	6	6	6

Note. Each cell in columns 2–4 shows the class number (1–24) and its assigned arm in parentheses. Class numbers within each arm are sequential (Arm A: classes 1–6; Arm B: classes 7–12; Arm C: classes 13–18; Arm D: classes 19–24). Every arm is covered by six of the eight teachers under the 24-of-32 cell-coverage allocation (Arm A: T1–T6; Arm B: T1, T2, T4, T6, T7, T8; Arm C: T1, T3, T4, T5, T7, T8; Arm D: T2,

T3, T5, T6, T7, T8), with all eight teachers contributing to three arms each. The total counts (6 classes per arm × 4 arms = 24 classes) match the participant flow in Table 2 of the main text.

S2.3 Arm-specific competence-assessment outcomes

Following the 32-h preparation workshop and prior to the main intervention, each of the eight teachers completed arm-specific competence assessments for the arms to which they were potentially assignable. Each assessment was scored on a 5-point rubric (assignment-design fidelity, feedback-protocol comprehension, gamification-mechanic familiarity, classroom-observation protocol comprehension, ethics-protocol compliance), with passing threshold $M \geq 3.50$. All eight teachers passed assessments for the three arms to which they were ultimately assigned. The mean competence-assessment score across all teacher-arm pairings was 4.21 ($SD = 0.34$, range 3.62–4.85). Detailed assessment scores by teacher-arm pairing are available from the corresponding author upon request.

S3. Illustrative Feedback Examples for Arm A (AI) and Arm B (Human Teaching Assistant)

S3.1 Purpose

This section responds to the distinction between matched feedback dose and feedback quality. Although Arms A and B were strictly matched on the four dose dimensions of frequency, dimension count, word-count range, and timing (Methods 3.2), equal dose does not guarantee equivalence in qualitative properties such as tone, contextual sensitivity, and responsiveness to a learner's expressed intention. The constructed examples assembled here—which are illustrations of feedback form and style rather than transcripts of individual student records—convey the shared five-dimension structure common to both arms alongside the residual stylistic differences between the automated and human sources, giving readers a concrete sense of the form the feedback took in each condition.

S3.2 Shared feedback protocol and template

Both arms delivered process feedback weekly, returned within 48 h of submission, within a 200–400-character range, and organized under five fixed dimensions: color harmony, luminance contrast, color-emotion expression, composition and balance, and technical execution. The two arms differed only in the feedback source.

- Arm A. Feedback was generated by ERNIE 4.0 via Baidu AI Cloud's enterprise API, with the model version fixed at ERNIE-4.0-8K-0613 throughout the intervention; generation parameters were temperature = 0 and top-p = 1, and each rating was an independent context-free call returning structured JSON output under the five-dimension template (Methods 3.3).
- Arm B. Feedback was written by trained human teaching assistants (24 h of feedback-writing training) using the same five-dimension template, delivering process feedback only and taking no part in classroom teaching, terminal grading, or the points system (Methods 3.2).

S3.3 Excerpt selection procedure

To enable a like-for-like comparison, the examples below were constructed by the authors to represent the typical form and stylistic character of the feedback delivered in each arm under the shared five-dimension template; they are not transcripts of any individual student's feedback record. Both examples are written for a single hypothetical assignment so that the AI and human sources can be compared on identical content, and the English is presented alongside the Chinese in which feedback was delivered to students, consistent with the bilingual presentation of the credibility-belief scale in Supplementary S5. The underlying archived feedback records are available from the corresponding author on reasonable request.

Illustrative scenario. A watercolor color-composition assignment on the theme "dusk" with an orange–blue contrasting palette. A single hypothetical work is used so that the Arm A and Arm B examples below address identical content.

S3.4 Illustrative excerpts by dimension

Table S3. *Constructed illustrative feedback examples for Arm A and Arm B across the five feedback dimensions*

Feedback dimension	Arm A — ERNIE 4.0 (AI) illustrative example	Arm B — human teaching assistant illustrative example
**Color harmony**	色彩和谐：作品以橙与蓝为主的对比配色，整体协调度中等。建议适度降低背景饱和度，以增强主体的视觉统一性。<br>*Color harmony: The work uses a predominantly orange–blue contrasting scheme; overall coherence is moderate. Lowering background saturation is recommended to strengthen the visual unity of the focal subject.*	你想用橙蓝对比表现暮色的张力，这个方向是对的。不过右上角那块高饱和蓝有点抢，主体反而被压下去了——把它往灰里调一点，画面会更聚。<br>*Your aim of using orange–blue contrast to convey the tension of dusk is the right instinct. The highly saturated blue in the upper right competes a little and pushes the subject back—muting it toward grey will pull the composition together.*
**Luminance contrast**	明度对比：明暗层次较为清晰，主体与背景的明度差约为中高水平。建议在中间调区域增加过渡层级，避免明暗断层。<br>*Luminance contrast: Tonal layering is fairly clear, with a moderate-to-high luminance difference between subject and background. Adding intermediate tonal steps in the midtone region would avoid abrupt transitions.*	明暗拉得开，远看很有力量。就是中间调有点空，眼睛从亮到暗跳得太快；可以在交界处补两三笔灰过渡，节奏会更稳。<br>*The light–dark range reads powerfully from a distance. The midtones feel a bit empty, so the eye jumps from light to dark too quickly; two or three grey transitional strokes at the boundary would steady the rhythm.*
**Color-emotion expression**	色彩情感表达：配色传达出一定的沉静与暖意，与主题基本契合。建议通	能感觉到你想表达那种黄昏将尽、有点惆怅的情绪，暖橙用得很到位。如

Feedback dimension	Arm A — ERNIE 4.0 (AI) illustrative example	Arm B — human teaching assistant illustrative example
	过局部冷暖对比强化情绪焦点。<br>*Color-emotion expression: The palette conveys a degree of calm and warmth, broadly consistent with the theme. Reinforcing an emotional focal point through localized warm–cool contrast is recommended.*	果想让"惆怅"更明确，可以让蓝调再低沉一点，别太鲜亮，情绪会更准。<br>*I can feel the wistful, day's-end mood you're after, and the warm orange serves it well. To make that wistfulness clearer, let the blues sink a little—less brightness—and the emotion will land more precisely.*
**Composition and balance**	构图与平衡：主体位置偏左，画面重心略失平衡。建议依据三分法调整主体位置，或在右下区域增加视觉元素以恢复平衡。<br>*Composition and balance: The subject sits left of center and the visual weight is slightly unbalanced. Repositioning the subject by the rule of thirds, or adding a visual element in the lower-right region, would restore balance.*	主体靠左其实挺好，有种留白的呼吸感；但右下角太空了，整张画往左倒。不一定要加东西，把右边那道光延长一点点也能压住。<br>*Placing the subject to the left actually works—it gives an open, breathing feel; but the lower-right is too empty and the whole image tips leftward. You don't necessarily need to add an object—extending that streak of light on the right a touch could anchor it.*
**Technical execution**	技法表现：用色较为均匀，边缘处理基本到位。建议在湿接处理上加强练习，以减少局部水痕。<br>*Technical execution: Color application is fairly even and edge handling is largely adequate. Further practice on wet-in-wet joins is recommended to reduce localized watermarks.*	上色整体干净，进步明显。就是天空那块湿接的时候水分没控好，留了点水痕——下次接的时候纸别太湿，等半干再接就稳了。<br>*Your application is clean overall and the improvement is clear. The water control slipped on the wet-in-wet join in the sky, leaving a watermark—next time let the paper reach the half-dry stage before joining and it'll hold.*

Note. These examples are constructed illustrations of the feedback format and stylistic character of each arm; they are not verbatim extracts of any student's records. The same five-dimension template governed both arms in the trial.

S3.5 Observed qualitative differences

Across the archived corpus, the two sources shared the same five-dimension scaffold but differed in stylistic surface. AI-generated feedback exhibited uniform structuring, fixed templating, and consistent dimensional coverage with limited tonal variation, properties consistent with large-language-model output under temperature = 0. Human teaching-assistant feedback preserved the five-dimension structure while showing greater contextual sensitivity and tonal variation, including occasional reference to a learner's evident intention. This characterization is qualitative and descriptive; it parallels the interpretation offered in Section 5.4 of the main text and is not intended as a quantitative comparison of feedback quality.

In the illustrative example above, for instance, the AI source names the affect generically and appends a templated recommendation, whereas the teaching assistant identifies the intended “wistful” mood and ties the suggestion to lowering blue brightness, illustrating the contextual-sensitivity difference noted above.

S4. T0 → T1 → T2 within-intervention mediation

S4.1 Purpose and methodological positioning

The T0 → T1 → T2 within-intervention mediation model is presented here as a methodological reference and as a contrast against the primary T0 → T1 → T3 longitudinal mediation model reported in the main text (Table 7, Figure 4). It is not intended as a substitute primary model. The within-intervention model measures the outcome Y at T2 (week 14, end of intervention), while the intervention X is still being administered; the indirect effect estimates from this model therefore capture within-intervention dynamic correlation rather than satisfying the longitudinal mediation causal-identification conditions of Cole and Maxwell (2003) and Maxwell et al. (2011).

The within-intervention model is presented for two reasons: (a) to allow readers and reviewers to compare the magnitude of indirect-effect estimates under the temporally inadequate within-intervention specification versus the temporally adequate post-intervention specification (Section 5.2); and (b) to provide a transparent record of all parallel models fitted during the analysis stage, in line with the open-science commitments of the present study.

S4.2 Model specification

The T0 → T1 → T2 within-intervention mediation model uses the identical autoregressive specification as the primary T0 → T1 → T3 model (Methods 3.4) but substitutes T2 outcome measures for T3 outcome measures. Specifically:

• Mediator (M): T1 MAI cognitive-regulation subscale (6 items, week 9), as in the primary model.

• Motivation outcome (Y_1): T2 MSLQ 15-item motivation composite (week 14). Because the primary T3 motivation outcome was 15 items, the T2 measurement substitutes the same 15-item instrument administered four weeks earlier at the end of intervention; note that T2 instrumentation in the main protocol was MAI + engagement only (Methods 3.3), so this T2 motivation measurement was administered as an additional measurement wave for sensitivity purposes only.

• Achievement outcome (Y_2): T2 color-work achievement (3 expert raters, 5-dim. rubric), substituting the T3 achievement instrument administered four weeks earlier.

• Covariates: T0 metacognitive regulation, T0 MSLQ, T0 color-knowledge pretest, demographic controls, teacher dummies (identical to primary model).

• Estimator: MLR with TYPE = COMPLEX and cluster-robust SEs.

• Indirect effects: Monte Carlo confidence intervals with 20,000 resamples.

S4.3 Within-intervention model results

Path coefficients and indirect effects are reported in Table S4 below. Compared with the primary T0 → T1 → T3 model:

• T0 → T1 autoregressive path β_AR was 0.582 in the primary model and 0.591 in the within-intervention model (essentially unchanged, as expected, since T1 mediator measurement is identical in both models).

• X → T1 (a path) was 0.224 (primary) and 0.224 (within-intervention model)—identical, as expected (both models use the same T1 mediator outcome).

• T1 M → Y motivation (b_1) was 0.371 (primary, T3 outcome) and 0.464 (within-intervention, T2 outcome); the larger T2 coefficient reflects the temporal proximity of T1 mediator measurement (week 9) and T2 motivation measurement (week 14), an interval of only 5 weeks during which X was still being administered.

• T1 M → Y achievement (b_2) was 0.286 (primary, T3 outcome) and 0.366 (within-intervention, T2 outcome); same pattern.

• Motivation-path indirect effect ab_1 was 0.083 (95% CI [0.039, 0.135], primary) and 0.104 (95% CI [0.054, 0.158], within-intervention). The within-intervention estimate is 25.3% larger than the primary estimate.

• Achievement-path indirect effect ab_2 was 0.064 (95% CI [0.025, 0.107], primary) and 0.082 (95% CI [0.037, 0.130], within-intervention). The within-intervention estimate is 28.1% larger than the primary estimate.

S4.4 Interpretation

The inflated within-intervention estimates relative to the post-intervention estimates are consistent with the methods-stage expectation that within-intervention dynamic correlation will exceed true longitudinal mediation (Section 5.2). When Y is measured while X is still being administered, the b-path captures both genuine M → Y transmission and concurrent ongoing X → Y contributions that have not yet had time to dissipate; the indirect-effect estimate is therefore upward-biased relative to the true longitudinal mediation parameter.

This pattern provides empirical support for the methodological choice to position T3 four weeks after intervention cessation as the primary outcome time point. The smaller but causally cleaner primary estimates (ab_1 = 0.083; ab_2 = 0.064) constitute the appropriate inferential basis for the H3 hypothesis.

S4.5 Table S4

Table S4. *T0 → T1 → T2 within-intervention mediation, parallel to Table 7*

Path	Estimate (T0→T1→T2)	95% CI	Estimate (T0→T1→T3)	95% CI
β_AR (T0 M → T1 M)	0.591	[0.530, 0.647]	0.582	[0.521, 0.638]
a (X → T1 M)	0.224	[0.128, 0.319]	0.224	[0.128, 0.319]
b_1 (T1 M → Y motivation)	0.464	[0.342, 0.586]	0.371	[0.265, 0.476]
b_2 (T1 M → Y achievement)	0.366	[0.241, 0.491]	0.286	[0.176, 0.394]
ab_1 Motivation indirect	0.104	[0.054, 0.158]	0.083	[0.039, 0.135]
ab_2 Achievement indirect	0.082	[0.037, 0.130]	0.064	[0.025, 0.107]
c'_1 Motivation direct	0.142	[0.054, 0.226]	0.118	[0.029, 0.205]
c'_2 Achievement direct	0.108	[0.022, 0.193]	0.091	[0.008, 0.176]

Note. All coefficients are standardized regression coefficients (β). Monte Carlo confidence intervals based on 20,000 resamples. T0→T1→T2 model uses end-of-intervention outcome measurement (week 14); T0→T1→T3 model uses post-intervention outcome measurement (week 18). The within-intervention model violates the longitudinal mediation causal-identification condition that X must have ceased before Y is measured (Cole & Maxwell, 2003; Maxwell et al., 2011); estimates from this model should be interpreted as descriptive within-intervention dynamic correlation, not as causal mediation.

S5. Prior AI credibility belief 4-item scale

S5.1 Source and adaptation

The prior AI credibility belief scale used in the present study is a 4-item adaptation of two subscales from Lankton et al. (2015), "Technology, humanness, and trust: Rethinking trust in technology," *Journal of the Association for Information Systems*, 16(10), 880–918. The original Lankton et al. (2015) instrument contains multiple subscales operationalizing trust in technology; we adapted the functionality subscale and the reliability subscale, which jointly correspond most directly to the construct of "learners' prior beliefs about the credibility of AI systems for educational tasks."

The 4-item adaptation passed an expert content-validity review (CVI = 0.83) and achieved Cronbach's α = 0.81 in the pilot sample (n = 46) and α = 0.84 in the main-trial T0 sample (N = 540). Item-total correlations in the main sample ranged 0.61–0.78.

S5.2 Item-selection rationale

From each subscale, items were retained on the basis of three a priori criteria, in descending order of weight:

• **Generality across AI use contexts.** Items referring to a specific application or domain (e.g., "this online recommendation agent") were not retained; only items referring to AI technology generally were retained, to align with the construct measured at T0 prior to the educational intervention.

• **Conceptual centrality to the subscale construct.** Items that most directly captured the subscale's defining construct (functionality = AI's task-execution capability; reliability = AI's consistency in producing dependable outputs) were preferred over items emphasizing secondary aspects.

• **Clarity in Chinese translation.** Items with the least translation ambiguity from English to Mandarin were preferred; two independent bilingual research assistants forward- and back-translated each candidate item, and only items with high translation concordance were retained.

This selection procedure produced a final 4-item scale with two items from the functionality subscale and two items from the reliability subscale.

S5.3 Final 4-item adapted scale (English)

Instructions: "For each statement below, please indicate how much you agree, considering AI technology in general (not any specific AI tool you may have used). Please respond on a 5-point scale: 1 = strongly disagree; 2 = disagree; 3 = neither agree nor disagree; 4 = agree; 5 = strongly agree."

Functionality subscale items (2 items):
F1. AI technology has the functionality I need to complete tasks.

F2. AI technology has the features required to perform what I expect.

Reliability subscale items (2 items):
R1. AI technology consistently performs the tasks it is designed to do.

R2. AI technology operates reliably and dependably.

S5.4 Chinese translation (used in the main trial)

指导语: "请根据您对人工智能技术的整体认识 (而非任何具体使用过的 AI 工具), 表明您对以下每一陈述的同意程度。请使用 1–5 分制评分: 1 = 非常不同意; 2 = 不同意; 3 = 既不同意也不反对; 4 = 同意; 5 = 非常同意。"

功能性 (Functionality) 题项 (2 题):
F1. 人工智能技术具备我完成任务所需的功能。

F2. 人工智能技术拥有执行我期望事项所需的特性。

可靠性 (Reliability) 题项 (2 题):
R1. 人工智能技术能够一致地完成它被设计执行的任务。

R2. 人工智能技术运行稳定可靠。

S5.5 Scoring

The composite prior AI credibility belief score is the mean of the four item ratings. Possible range: 1.00–5.00. In the main-trial T0 sample (N = 540), composite-score M = 3.45, SD = 0.74, range 1.00–5.00.

S5.6 Limitations of the 4-item adaptation

The 4-item adaptation inevitably sacrifices construct coverage relative to the original full subscales of Lankton et al. (2015). Limitations explicitly acknowledged in Section 5.5 of the main text: (a) reduced number of items per subscale limits internal-consistency stability and potentially attenuates discriminant validity against neighboring constructs (e.g., general technology acceptance); (b) the helpfulness subscale of Lankton et al. (2015), which captures AI's responsiveness to user needs, is not represented in the 4-item version. Future studies in larger samples should employ the full Lankton et al. (2015) subscales without item reduction.

Table S5. Baseline equivalence sensitivity references and H1/H2a/H2b sensitivity-analysis results

Table S5 is referenced at two points in the main text (Results 4.1 baseline equivalence and Results 4.4 main-effects sensitivity). The table is organized in two panels.

Panel A. Baseline equivalence: unadjusted vs. covariate-adjusted models

Two baseline variables showed marginal between-arm differences in the unadjusted cluster-robust ANCOVAs reported in Table 5 of the main text: prior color-course experience (p = .087; Arm D slightly lower than other three arms) and T0 learning motivation (p = .043; Arm C slightly higher than Arm D). Both were addressed in the primary analyses through covariate adjustment in the H1, H2a, H2b, H3, and H4 models. Panel A documents the marginal between-arm contrast under both adjusted and unadjusted models for these two variables.

Table S5, Panel A. *Baseline equivalence under adjusted vs. unadjusted models*

Variable	Arm A	Arm B	Arm C	Arm D
Prior color-course exp. (1–5)	2.91	2.85	2.87	2.64
unadjusted contrast: ANOVA F = 2.21, p = .087				
adjusted contrast (covariates): F = 1.43, p = .234				
T0 learning motivation (1–5)	3.69	3.66	3.81	3.62
unadjusted contrast: ANOVA F = 2.74, p = .043				
adjusted contrast (covariates): F = 1.78, p = .151				

Note. Both marginal baseline differences attenuated to non-significance after adjustment for the full covariate set (T0 same-name variables, demographic variables, teacher dummies). The primary analyses' use of covariate adjustment therefore handles the two baseline differences appropriately. The remaining eight baseline variables (Table 5) were equivalent across arms in both unadjusted and adjusted models (p

= .13–.91 in unadjusted; all p > .20 in adjusted).

Panel B. H1/H2a/H2b results across three sensitivity-analysis schemes

In Section 4.4 of the main text, three sensitivity-analysis schemes were used to evaluate the robustness of the H1, H2a, and H2b main-effects conclusions to specification choices regarding cluster-correction method. The three schemes were:

• **Scheme A (primary, wild cluster bootstrap):** MLR with TYPE = COMPLEX cluster-robust SEs, supplemented by wild cluster bootstrap (Cameron et al., 2008; 5,000 resamples; 6-point Webb [2023] distribution). This is the specification reported in Table 7 of the main text.

• **Scheme B (class fixed-effects dummy):** Cluster-robust ANCOVA replaced by a model in which each class is represented by an indicator variable, with cluster-robust SEs replaced by HC3-corrected SEs. This specification consumes one degree of freedom per class, with $G - 1 = 23$ class dummies in total.

• **Scheme C (ignoring nesting):** Standard ANCOVA without cluster correction; this scheme is presented as a transparency reference to document the magnitude of SE underestimation produced by ignoring the class-level nesting.

Table S5, Panel B. *H1/H2a/H2b under three sensitivity specifications*

Hypothesis	Scheme A (wild cluster bootstrap, primary)	Scheme B (class fixed-effects dummy)	Scheme C (ignoring nesting; reference only)
H1 (C vs. D)	d = 0.34, p = .005, p_Holm = .015	d = 0.32, p = .008, p_Holm = .024	d = 0.37, p < .001, p_Holm < .003
H2a (A vs. B)	d = 0.07, p = .555, p_Holm = .555	d = 0.06, p = .603, p_Holm = .603	d = 0.07, p = .463, p_Holm = .463
H2b (A+B vs. C)	d = 0.24, p = .024, p_Holm = .048	d = 0.20, p = .062, p_Holm = .062	d = 0.27, p = .003, p_Holm = .009

Note. Scheme A is the primary specification reported in the main text and Table 7. Scheme B yielded a non-significant H2b result (p = .062), indicating that the principal H2b conclusion is specification-sensitive to the choice of cluster-correction method. The H2b conclusion in the main text is therefore appropriately characterized as marginal and specification-sensitive (Section 4.4 and Section 5.1). Scheme C demonstrates the magnitude of SE underestimation when nesting is ignored: H1 and H2b p-values are systematically smaller than under Schemes A and B, illustrating why cluster-robust inference is preferred.

Sensitivity-analysis summary on H1, H2a, H2b: H1 conclusion is robust across all three schemes (d = 0.32–0.37, p_Holm < .025 in all schemes); H2a non-significance is robust across all three schemes (d ≈

0.07 in all schemes); H2b conclusion is specification-sensitive, retaining significance under Schemes A and C but attenuating to non-significance under Scheme B. The principal H2b conclusion is therefore characterized as marginal and specification-sensitive throughout the main text.
